# An intervention modelling experiment to change GPs' intentions to implement evidence-based practice: *using theory-based interventions to promote GP management of upper respiratory tract infection without prescribing antibiotics #2*

**DOI:** 10.1186/1472-6963-8-10

**Published:** 2008-01-14

**Authors:** Susan Hrisos, Martin Eccles, Marie Johnston, Jill Francis, Eileen FS Kaner, Nick Steen, Jeremy Grimshaw

**Affiliations:** 1 Institute of Health and Society, Newcastle University, UK; 2 Department of Psychology, University of Aberdeen, UK; 3 Health Services Research Unit, University of Aberdeen, UK; 4 Clinical Epidemiology Program, Ottawa Health Research Institute and Department of Medicine, University of Ottawa, Ottawa, Canada

## Abstract

**Background:**

Psychological theories of behaviour may provide a framework to guide the design of interventions to change professional behaviour. Behaviour change interventions, designed using psychological theory and targeting important motivational beliefs, were experimentally evaluated for effects on the behavioural intention and simulated behaviour of GPs in the management of uncomplicated upper respiratory tract infection (URTI).

**Methods:**

The design was a 2 × 2 factorial randomised controlled trial. A postal questionnaire was developed based on three theories of human behaviour: Theory of Planned Behaviour; Social Cognitive Theory and Operant Learning Theory. The beliefs and attitudes of GPs regarding the management of URTI without antibiotics and rates of prescribing on eight patient scenarios were measured at baseline and post-intervention. Two theory-based interventions, a "graded task" with "action planning" and a "persuasive communication", were incorporated into the post-intervention questionnaire. Trial groups were compared using co-variate analyses.

**Results:**

Post-intervention questionnaires were returned for 340/397 (86%) GPs who responded to the baseline survey. Each intervention had a significant effect on its targeted behavioural belief: compared to those not receiving the intervention GPs completing Intervention 1 reported stronger self-efficacy scores (Beta = 1.41, 95% CI: 0.64 to 2.25) and GPs completing Intervention 2 had more positive anticipated consequences scores (Beta = 0.98, 95% CI = 0.46 to 1.98). Intervention 2 had a significant effect on intention (Beta = 0.90, 95% CI = 0.41 to 1.38) and simulated behaviour (Beta = 0.47, 95% CI = 0.19 to 0.74).

**Conclusion:**

GPs' intended management of URTI was significantly influenced by their confidence in their ability to manage URTI without antibiotics and the consequences they anticipated as a result of doing so. Two targeted behaviour change interventions differentially affected these beliefs. One intervention also significantly enhanced GPs' intentions not to prescribe antibiotics for URTI and resulted in lower rates of prescribing on patient scenarios compared to a control group. The theoretical frameworks utilised provide a scientific rationale for understanding how and why the interventions had these effects, improving the reproducibility and generalisability of these findings and offering a sound basis for an intervention in a "real world" trial.

**Trial registration:**

Clinicaltrials.gov NCT00376142

## Background

The consultation for upper respiratory tract infection (URTI) is one of the commonest in general practice [1]. Systematic reviews have shown that antibiotics are of limited effectiveness in the treatment of URTI [2,3]. Growing public health concerns about the implications of overprescribing of antibiotics for antimicrobial resistance in general have brought doctors under pressure to reduce inappropriate prescribing of these drugs [4-6]. Major public health campaigns have also targeted patients to deter consultation for URTI. Encouragingly, recent years have seen both a decline in the number of patients consulting for URTI [7,8] and the prescribing of antibiotics for URTI in UK general practice [9]. Between 1993 and 2001, prescribing reduced nationally by 30% for coughs and colds and by 47% for sore throat. However there remained substantial regional variation in practice[9] and general practitioners (GPs) continued to prescribe for up to 42% patients presenting with uncomplicated URTI.

Such varied uptake of research evidence into routine practice may be due to the currently imperfect evidence-base to guide the choice and design of effective interventions to change professional behaviour [10]. Few implementation studies provide an underlying theoretical basis to explain how or why successful interventions work or have been preceded by exploratory studies to test the feasibility of or to refine an intervention. We aimed to address this gap in the current implementation evidence-base through the application of a systematic, theory-based intervention modelling process (IMP) for the development and evaluation of interventions to change clinical practice [11]. The process involves stages of development – currently lacking in implementation research – that closely correspond to the theoretical, modelling and experimental phases of the MRC Framework for the evaluation of complex interventions [12].

### Using theory to develop and evaluate implementation interventions

We used the IMP in the design of the content of two theory-based interventions to change the behaviour of GPs with respect to their management of URTI. Detailed description of the interventions and their systematic development is provided in our partner paper [13]. As part of this initial stage of the IMP, three psychological theories of behaviour change were selected as the framework to inform the content of the interventions: the Theory of Planned Behaviour (TPB) [14]; Social Cognitive Theory (SCT) [15] and Operant Learning Theory (OLT) [16]. These theories all explain behaviour in terms of factors that are amenable to change. They each provided theoretical constructs (e.g. beliefs, attitiudes) that were found to be the antecedents of both GPs' intended and actual behaviour in relation to their management of URTI. The interventions were designed to differentially target these important constructs by incorporating relevant and evidence-based behaviour change techniques.

In addition to guiding the design of implementation interventions, the selected theories also provide a means to experimentally evaluate and refine implementation interventions. As these theories identify the proximal predictors of behaviour they provide measurable interim endpoints that represent actual behaviour (usually intention). They also identify beliefs that can mediate the effect of an intervention on this proxy measure of behaviour. The potential effect of an intervention on actual behaviour and the underlying behavioural process that drives it can thus be examined by conducting an intervention modelling experiment (IME). IMEs have been used successfully in the evaluation of interventions targeting health behaviours in patient populations, with effects replicated in "real world" studies [17-19]. We have further demonstrated the utility of this method in relation to clinicians' behaviour [20,21].

This paper reports the experimental evaluation of the two theory-based interventions using this method, and which forms an additional stage of the IMP. The evaluation was designed to answer the following research questions:

Do the theory-based interventions influence GPs' behavioural intention and/or their simulated behaviour in the management of URTI without prescribing antibiotics?

Do the theory-based interventions influence the targeted theoretical constructs?

## Methods

### Design and participants

The design was a randomised 2 × 2 factorial randomised controlled trial with baseline and post-intervention assessment. The theoretical frameworks used for this evaluation were the same as those used in the development of the theory-based interventions [11]. Measures were delivered in two postal questionnaire surveys, with the interventions incorporated at the start of the second questionnaire booklet (Additional file 1). Participants responding to the first survey were included in the second. Randomisation was at the level of general practice to ensure that participants within each practice received the same intervention and was stratified by the number of baseline respondents per practice to ensure equivalence of groups. Practices were randomised twice to receive, or not, each of two study interventions, producing four comparison groups: no intervention; Intervention1, Intervention 2; both intervention 1 & 2. The study participants were all of the general practitioners (GPs) serving 13 Primary Care Trusts in the North East region of the UK.

### Outcome Measures

#### Behavioural Intention

This was measured using the standard methods used in investigations based on the Theory of Planned Behaviour i.e. using rating scales of likelihood, frequency or agreement with statements or questions about behavioural intention [14,22]. Four items assessed behavioural intention to manage a patient with an URTI without prescribing an antibiotic: *When a patient presents with an URTI, I have in mind to manage them without prescribing an antibiotic; I intend to manage patients who present with an URTI without prescribing an antibiotic; I aim to manage patients who present with an URTI without prescribing an antibiotic (all scored on a scale 1 "Strongly Disagree" to 7 "Strongly Agree"); Given 10 patients presenting for the first time with the an URTI, how many patients would you intend to manage without prescribing an antibiotic? (scored on the scale 0 to 10) *[Additional file 1; section 1]. The behavioural intention score was the sum of the 4 variables (as the scales differed, behavioural intention scores were converted to z-scores and summed). Higher behavioural intention scores reflected a stronger behavioural intention to manage URTI without prescribing an antibiotic.

#### Behavioural simulation

Two sets of eight patient scenarios were developed, one set for each postal survey. Each required the respondent to simulate the behaviour they would enact in the real clinical situation. The scenarios reflected the range of patients and clinical features that present in general practice informed by qualitative work conducted in a previous study [23]. Features identified in this study as influencing GPs' choice of management strategy were systematically allocated between the two sets of scenarios. The presentation format of the scenarios was designed to replicate the presentation of patient information as seen by a GP on the surgery computer screen [Additional file 1; section 2]. The format also included a simulated prescription pad, free text sections to note the diagnosis and management. The behavioural simulation score was the total number out of eight scenarios for which antibiotics were not prescribed.

### Process (explanatory) measures

The process measures used in the postal questionnaire were derived from semi-structured elicitation interviews with 14 GPs in Scotland [23]. The interviews covered doctors' views and experiences relating to the management of an URTI. Responses were coded into belief domains (behavioural, normative, control) which were then used, in conjunction with the literature, to create questionnaire items measuring variables from the psychological theories on a 7-point scale from *Strongly Disagree to Strongly Agree *[Additional file 1; section 1]. Theoretically derived measures follow the operationalisation protocols of Ajzen [14], Bandura [15] and Francis *et al.*[22]. Table 1 provides a summary of the measures used in this study.

**Table 1:** Summary of the theoretical constructs used as predictive measures

**Variables (number of questions)**	**Example Item(s)**
***Theory of Planned Behaviour (TPB) (Ajzen, 1991)***	

Behavioural intention (3 & 4). Two summary scores: sum of three and four items	I intend to manage patients with URTIs without prescribing an antibiotic (scored 1 to 7) Given 10 patients presenting for the first time with an URTI, how many patients would you intend to manage without prescribing an antibiotic? (Scored 1 to 10)
Attitude: Direct (3); Indirect (8 behavioural beliefs (bb) multiplied by 8 outcome evaluations (oe). The score was the mean of the summed multiplicatives.)	*Direct*: In general: The benefits of managing patients with URTI without prescribing antibiotics outweighs the harms; *Indirect*: In general, managing a patient with an URTI without prescribing an antibiotic would reassure them (bb) × reassuring the patient is (oe: un/important)
Subjective Norm: I (5 normative beliefs (nb) multiplied by 5 motivation to comply (mtc) items. The score was the mean of the summed multiplicatives).	I feel under pressure to manage patients with an URTI without prescribing an antibiotic: from published literature (nb) × How motivated are you to do what the published literature states that you should (mtc: very much/not at all)
Perceived Behavioural Control: Direct (4) Indirect (6)	*Direct*: Whether I manage patients with an URTI without prescribing an antibiotic is entirely up to me *Indirect*: I find it difficult to manage patients presenting with an URTI without prescribing an antibiotic who: Expect me to prescribe an antibiotic

***Social Cognitive Theory (SCT) ****(Bandura,1997)*	

Risk Perception (3)	It is highly likely that patients with an URTI will be worse off if I manage them without prescribing an antibiotic.
Outcome Expectancies: Behaviour (8 × 8) The score was the mean of the summed multiplicatives.	*Behaviour*: See Attitude (Theory of Planned Behaviour)
Self Efficacy: Specific (6)	*Specific*: Without an antibiotic: How confident are you in your ability to manage patients with URTIs who have tried to self-medicate

***Operant Learning Theory (OLT) ***[16]	

Anticipated consequences (3)	If I routinely manage patients with URTIs without prescribing an antibiotic then, on balance, my life as a GP will be easier in the long run
Evidence of habit (2)	When I see patients with URTIs, I automatically consider managing them without prescribing an antibiotic

***Additional measures***	

Implementation Intention (Gollwitzer, 1993)	
Prior planning (1)	Currently, my standard method of managing patients with an URTI involves managing them without prescribing an antibiotic
Action planning (3)	I have a clear plan of how to manage patients with an URTI without prescribing an antibiotic I have a clear plan of when to manage patients with an URTI without prescribing an antibiotic I have a clear plan of under what circumstances to manage patients with an URTI without prescribing an antibiotic
*Other Measures*	
Demographics	gender, years qualified, trainer status, single or multi-practitioner practice

Three additional measures were included in the questionnaire: the extent of "prior planning" and "action planning" from the Implementation Intention model (II) [24] and "evidence of habit" from OLT. These constructs have been found to be significant predictors of GP behaviour in relation to the management of URTI [23]. In the II model "prior planning" and "action planning" represent the extent to which an individual has developed an explicit plan, or an "implementation intention, about when and where an intended action or "goal behaviour" will be achieved. An implementation intention is therefore a "post-intentional" variable. As one of the interventions includes an element of "making a plan", the extent of GPs' prior planning was assessed by a single item (Additional file 1, Section 1, Item 18) and "action planning" by three items (Additional file 1, Section 1, Items 19a, b & c).

OLT proposes that repeated behaviours may become "habitual". Habitual behaviour is a strong predictor of behaviour but could be described more accurately as an attribute of behaviour rather than a causal determinant. Change in habit, or the formation of new habits, is influenced indirectly by targeting factors that are causally related to the behaviour. As a general aim of both study interventions was to promote the habit of managing patients with URTI without an antibiotic, a measure of "evidence of habitual behaviour" was included in the questionnaire. Two items assessed evidence of habitual behaviour (Additional file 1, Section 1, Items 7a & b).

### Interventions

Two paper-based, behaviour change interventions were evaluated. The interventions are named according to the principle behaviour change technique used in their development [11].

#### Graded Task

This intervention targeted the theoretical construct of self-efficacy (SCT) using the behaviour change techniques of graded task; rehearsal; and action planning. The aim of this intervention was to increase the individual's belief in his/her capabilities to manage uncomplicated URTI without prescribing antibiotics. (Additional file 2).

#### Persuasive communication

This intervention targeted the theoretical constructs of anticipated consequences (OLT) and risk perception (SCT). [As these constructs overlap considerably in their description and their operationalisation, this intervention is referred to from here on as targeting "anticipated consequences". The distinction between the two constructs is maintained when discussing them in relation to their source theories]. The aim of this intervention was to influence the individual's beliefs about the positive consequences of managing URTI without prescribing antibiotics (Additional file 3).

### Intervention Fidelity

Participant engagement with the interventions was measured as the degree of completeness of the written tasks presented by each intervention. We considered that GPs who had completed at least one of the 3 parts of the Graded Task intervention had engaged interactively with this intervention. We also looked to see if the pattern of actual responses to part A ("Yes", "Maybe" and "No") followed the pattern we anticipated.

For the persuasive communication intervention, interactive engagement was assumed if the GP had completed one or both of the rating scales on this intervention.

### Procedure

#### Delivering the modelling experiment

The first postal questionnaire survey ran from 1^st ^September to mid-October 2005 and the second survey ran from mid-October to the end of November 2005. For the first survey all GPs received a letter of invitation, and the survey booklet (Additional file 1). The booklet included a set of instructions, questions addressing the process measures (behavioural antecedents and beliefs) and outcome measures (behavioural intention and behavioural simulation) and a set of eight patient scenarios. GPs were instructed to complete the different sections of the booklet in the order that they were presented. By responding to the first survey GPs consented to participate in the second survey. To increase response rates, a £20 incentive was offered to each participant who returned the first questionnaire [25,26].

Two months after the first mailing, respondents were randomised at the practice level to one of the four study groups and then mailed the second survey. For those GPs allocated to an intervention group, the survey booklet now also contained the appropriate interventions, in addition to the process and outcome measures. The interventions were always the first section of the survey booklet, and GPs were again instructed to complete each section in the order of presentation. On both occasions two reminders were mailed to non-responding clinicians.

### Sample size and Analysis

The experiment was powered to detect a difference between each of the active intervention groups and the control group. Using standard methods for a continuous outcome, 50 participants were required per group to have 80% power of detecting an effect size of 0.8 using a significance level of 2.5%, giving a total sample size of 200 for the experiment. We over-sampled to ensure achieving this final sample size, using an initial sample of 1225.

Data were analysed using SPSSPC [27]. For composite variables, where contributing items were missing these were replaced with the individual respondent's mean score of the remaining items in the composite set, providing that 50% of the measure was complete. Missing data for single item measures were not replaced. Internal consistency of multi-item measures was assessed using Cronbach's alpha (for measures with >2 items) using an acceptability criterion of α > 0.6, and Pearson's correlation coefficient (for 2 item measures) using an acceptability criterion of r > 0.25.

Relationships between explanatory (process) and outcome variables were examined using Pearson correlations and stepwise regression analyses. The trial groups were compared using methods appropriate for comparing independent samples (t-tests to compare two groups, analysis of variance to compare multiple groups and analysis of covariance to compare two or more groups adjusting for differences in baseline performance). When undertaking analysis of variance and analysis of covariance the first step was to fit a full factorial model. If the interaction between the two interventions was not significant it was removed from the model and the estimate of the effect of each intervention was based on a main effects model. The extent to which the antecedent beliefs mediated effects on the outcomes was tested using the Baron and Kenny methodology [28], and the Sobel test [29].

### Ethics approval

The study was approved by the Northern and Yorkshire Multi-Centre Research Ethics committee.

## Results

### Response rates and non-response analyses

**Figure 1:**
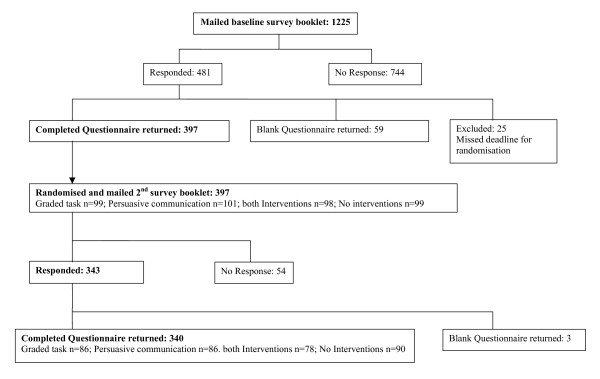
Response rates.

One thousand, two hundred and twenty-five GPs at 289 practices were sent the pre-intervention survey booklet. Six hundred and sixty-eight (60%) GPs were male (excludes 78 GPs where gender was not confirmed), and 245 (96%) practices were multi-practitioner surgeries. Completed questionnaires were returned by 397 (32.4%) GPs from 191(66%) practices (Figure 1). GPs responding to this first mailing had been qualified for a mean (SD) of 19.9 (8.0) years, 21% were GP trainers, 97% were from multi-practitioner surgeries and 56% were male. These 397 respondents were randomised to receive the study interventions and were mailed the post-intervention survey booklet. Three hundred and forty (86%) GPs returned the post-intervention survey booklet, from 178/191 (93%) practices. These 340 GPs had been qualified for a mean (SD) of 19.9 (8.1) years, 22% were GP trainers, 97% were from multi-practitioner surgeries and 56% were male.

#### Non-response analysis

The response rate to the first mailing was fairly consistent across the 13 PCTs (average PCT response rate (SD) = 39% (7.6)). The final sample of 340 GPs did not differ significantly from the original sampling frame of 1225 GPs on gender (chi-square = 2.916, df = 1, p = 0.09, excludes 78 where gender was not confirmed for non-respondents) or multi-practitioner practice status (chi-square = 0.001, df = 1, p = 0.971).

### Equivalence of intervention groups

The randomised groups did not differ significantly on any demographic variables (Gender: chi-square = 3.105, df = 3, p = 0.376; GP trainer: chi-square = 2.563, df = 3, p = 0.439; years qualified: F = 0.904, df = 3, p = 0.439) or on their pre-intervention behavioural intention and behavioural simulation scores (Intention: F = 0.839, df = 3, p = 0.473; Behavioural simulation: F = 0.252, df = 3, p = 0.860).

### Intervention Fidelity

Full completion of both interventions was high. Of 164 GPs allocated to receive the Graded Task intervention 158 (96%) completed it fully, 5 partially completed it and 1 did not complete the intervention materials at all. However, 25/158 (16%) selected the easiest situation in the list (situation 1), rather than the easiest situation from those they had rated as "Maybe" or "No", and based their action plan on this situation. In general, GPs graded the five situations in the order of difficulty that followed the expected pattern of the intervention gradation (i.e. Yes, Maybe, No) (Table 2). This gradation was consistent for 78% GPs. GPs were spread across the situations that they found the least difficult to resolve and thus chose as the basis for their management "plan". One hundred and sixty-one of 164 (98%) GPs completed the persuasive communication intervention, but 3/161 did not complete the comparison rating scales, so may not have engaged with the intervention. The spread of GP self-ratings on each of these scales is shown in Table 2. The difference between these paired ratings was significant.

**Table 2:** Intervention Fidelity

**Graded Task intervention**	**Description of situation**												**% GPs rating "Yes" (N = 163)**	**% GPs using situation as basis for their management "plan" (N = 158)**
**Situation 1**	*Could you confidently end a consultation for a patient with an URTI without prescribing an antibiotic?*												94	17
**Situation 2**	*Could you confidently manage patients with URTIs without an antibiotic, who have already tried to self-medicate?*												75	12
**Situation 3**	*Could you confidently manage patients with URTIs without an antibiotic, who expect you to prescribe an antibiotic?*												62	17
**Situation 4**	*Could you confidently manage patients with URTIs without an antibiotic, whose symptoms are distressing them*												45	17
**Situation 5**	*Could you confidently manage patients with URTIs without an antibiotic, who have a past history of COPD*^1^*?*												9	30
**Own situation**	*Where another doctor had already prescribed antibiotics for the same episode*												-	7

**Persuasive communication**	**Description of rating**	**Proportion of GPs rating themselves as 0–100% like Dr B^2 ^(N = 161)**												
		
		**0%**	**10%**	**20%**	**30%**	**40%**	**50%**	**60%**	**70%**	**80%**	**90%**	**100%**	**%Mean (SD)**	**Mean Difference (t-test)**

**Rating 1 **(Intended behaviour)	% GP "tries to be like Dr B^2^"	3	4	2	2	0	1	1	4	11	44	28	82.1 (25.6)	10.8 (t = 8.326, p < 0.001)
**Rating 2 **(Actual behaviour)	% GP is "actually like Dr B"	1	1	1	3	2	6	13	26	25	18	4	71.3 (18.5)	

^1^chronic obstructive airways disease. ^2^Dr B "manages patients with URTI symptomatically".

### Item Analysis

Cronbach's alpha for the summary variable of PBC direct was 0.55 when all four contributing items were included. Removing one item (*"I would like to manage patients with URTI without prescribing an antibiotic, but I don't really know if I can"*) improved the alpha for the summary measure to 0.64 (Table 3). Cronbach's alpha for the summary measure of Attitude direct (α = 0.55), was not improved by removing any of the three items contributing to this composite variable so all three items were retained.

**Table 3:** Post-intervention descriptive statistics, correlations of process variables with intention and behavioural simulation & regression analyses by theory.

**Model**	**Process (explanatory) variables**	**N items**	**Alpha**	**Mean (SD)**	***Behavioural intention***				***Behavioural simulation***			
					
					r*	***B***	***Beta***	***Model summary R2(adj)***	r*	***B***	***Beta***	***Model summary R2(adj)***
**TPB**	Attitude direct	3	0.55	16.9 (2.9)	0.456****	0.301****	0.267		0.23****			
	Attitude Indirect	8	-	205.6 (36.1)	0.364****	0.017****	0.195		0.08^ns^			
	Subjective Norm	5	-	97.2 (36.7)	0.226****	0.014***	0.157		0.07^ns^			
	PBC direct	3	0.64	11.9 (3.5)	0.082^ns^	-0.018^ns^	-0.020		0.108**	0.026^ns^	0.055	
	PBC indirect	6	0.79	26.3 (5.6)	0.388****	0.153****	0.266	0.33	0.319****	0.076****	0.258	
	Behavioural intention	4	0.83	0.01 (3.2)					0.307****	0.108****	0.210	0.14
**SCT**	Risk Perception (same as anticipated consequences)	3	0.61*	16.6 (2.8)	0.441****	0.344****	0.298		0.191****	0.029**	0.144	
	Outcome Expectancies (same as attitude indirect)	8	-	205.6 (36.1)	0.364****	0.017****	0.190		0.105**	-0.034^ns^	-0.087	
	Self Efficacy	6	0.88	29.2 (5.4)	0.411****	0.178****	0.294	0.32	0.391****	0.122****	0.391	0.17
**OLT**	Anticipated consequences	3	0.61	16.6 (2.8)	0.441****	0.257****	0.224		0.197****	0.026^ns^	0.102	
	Evidence of habit	2	0.61	11.4 (2.1)	0.746****	1.048****	0.671	0.60	0.294****	0.202****	0.251	0.10
Post-intentional variables												
**II**	Prior planning	1	-	5.8 (1.0)					0.343****	0.545****	0.332	0.12
	Action planning	3	0.92	16.5 (3.0)					0.216****	0.114****	0.206	0.05
**Cross-theory analysis **(variables retained in regression models)												
**TPB**	Attitude direct					0.102**	0.101					
	Subjective Norm					0.010***	0.117					
**SCT/OLT**	Risk Perception/Anticipated consequences					0.194****	0.168					
**OLT**	Evidence of habit					0.948****	0.605					
**SCT**	Self-efficacy					0.061**	0.090	0.63	0.393****	0.091****	0.294	
**II**	Prior planning								0.343****	0.326****	0.198	0.18

* = Pearson correlation coefficient. **p < 0.05. ***p = 0.001. ****p < 0.001. ns = non-significant

### Relationships between explanatory and outcome measures

Table 3 presents the post-intervention correlation and regression coefficients for relationships between process and outcome variables by theoretical framework and overall, for all respondents. With the exception of PBC direct, all TPB variables were significantly correlated with scores on the behavioural intention outcome measure. Attitude indirect and subjective norm did not correlate with behavioural simulation. All SCT and OLT variables were significantly correlated with both outcome measures. Past behaviour was significantly associated with both behavioural intention and behavioural simulation, while both post-intentional variables, prior planning and action planning, significantly correlated with behavioural simulation.

The TPB explained 33% of the variance in behavioural intention, SCT 32% and OLT 60%. For behavioural simulation, the TPB explained 14% of the variance, SCT 17% and OLT 10%. All constructs from each model were entered simultaneously into regressions on behavioural intention and behavioural simulation, and allowed to compete. Attitude direct, subjective norm, anticipated consequences, habitual behaviour and past behaviour significantly predicted behavioural intention. Together these variables explained 63% of the variance in behavioural intention. The strongest predictor in this cross-theory model was "evidence of habit" (*Beta *= 0.605, p < 0.001). Self-efficacy and prior planning were the only significant predictors of behavioural simulation, together explaining 18% variance (Table 3).

### Trial outcome

#### Did the theory-based interventions influence GPs' behavioural intention and/or their simulated behaviour in the management of URTI without prescribing antibiotics?

No significant interaction effect of receiving both interventions was observed, thus the main effects for each intervention are reported (Table 4). Behavioural simulation scores (Mean (sd) for pre-intervention and post-intervention respectively) were: Graded Task: received intervention (n = 164): 5.40 (1.49) & 5.10 (1.70), did not receive intervention (n = 176): 5.36 (1.43) & 4.97 (1.65); Persuasive communication: received intervention (n = 164): 5.35 (1.38) & 5.25 (1.59), did not receive intervention (n = 176): 5.41 (1.53) & 4.83 (1.73)

**Table 4:** Trial analysis: Effect size and 95% Confidence Intervals

Constructs	Covariate analysis: Estimates of Effect Size			
	
	Graded Task		Persuasive communication	
	Beta	95% CI	Beta	95% CI

**Behavioural intention**	0.33	-0.16, 0.82	**0.90**	**0.41, 1.38**
**Simulated behaviour**	0.10	-0.18, 0.38	**0.47**	**0.19, 0.74**
Attitude (direct)	-0.19	-0.75, 0.37	**0.68**	**0.12, 1.24**
Attitude (Indirect)/Outcome expectancies	-1.73	-8.6, 5.11	**14.60**	**7.79, 21.5**
Subjective Norm	-1.42	-7.55, 4.7	**7.61**	**1.44, 13.8**
PBC (direct)	-0.04	-0.69, 0.61	0.22	-0.43, 0.87
PBC (indirect)	**1.76**	**0.88, 2.64**	0.76	-0.12, 1.64
**Anticipated Consequences/Risk perception**	0.04	-0.48, 0.57	**0.98**	**0.46, 1.50**
**Self efficacy**	**1.44**	**0.64, 2.25**	**0.85**	**0.04, 1.66**
Habit	0.19	-0.19, 0.56	**0.49**	**0.07, 0.82**
Prior Planning	0.09	-0.08, 0.26	**0.20**	**0.03, 0.37**
Action Planning	0.17	-0.34, 0.69	0.28	-0.24, 0.79

##### Graded Task intervention

There was no significant effect of this intervention on either behavioural intention not to prescribe an antibiotic or on GPs' simulated behaviour.

##### Persuasive communication intervention

There was a significant effect of this intervention on behavioural intention and behavioural simulation. GPs receiving this intervention had, on average, intention scores that were 0.9 units higher than those who did not receive it, indicating that their intentions were stronger (Table 4). They also prescribed on 0.47 fewer patient scenarios.

#### Did the theory-based interventions influence their targeted theoretical constructs?

The Graded Task intervention had a significant main effect on the theoretical construct targeted by this intervention (self-efficacy) and also on the TPB construct of PBC (indirect) (Table 4). GPs who experienced this intervention had stronger beliefs in their capabilities to manage patients with URTI without antibiotics and a stronger sense of perceived behavioural control compared to GPs who did not experience this intervention. The persuasive communication had a significant main effect on the construct targeted by this intervention (anticipated consequences). A significant effect was observed on a number of constructs not specifically targeted by this intervention (Table 4).

#### Mediational analyses

Further investigation examined whether the effect of the Persuasive Communication on behavioural intention and behavioural simulation was mediated through anticipated consequences, or any of the non-targeted constructs. Mediational analyses showed that the effect of this intervention on behavioural intention was partially mediated through its targeted construct (anticipated consequences: Sobel test statistic = -3.59, p < 0.001). In addition, the effect of this intervention was also partially mediated through both measures of TPB attitude (Attitude direct: Sobel test statistic = -2.49, p = 0.01; Attitude indirect: Sobel test statistic = 3.17, p = 0.001), the TPB measure of subjective norm (Sobel test statistic = -2.36, p = 0.018) and the SCT measure of self-efficacy (Sobel test statistic = -1.98, p = 0.048).

The effect of the persuasive communication on behavioural simulation was again partially mediated through the targeted construct of anticipated consequences (Sobel test statistic = -2.53, p = 0.012). Mediation also occurred through TPB Attitude direct (Sobel test statistic = -2.20, p = 0.028) and the SCT measure of self-efficacy (Sobel test statistic = -1.97, p = 0.049)

## Discussion

The objective of the present study was to experimentally evaluate the effect of two theory-based interventions on the behavioural intention and simulated behaviour of GPs in relation to the management of uncomplicated URTI. We did this using a randomised controlled trial design, within the context of an Intervention Modelling Process (IMP). The interventions were designed to change beliefs previously identified as important predictors of antibiotic prescribing by GPs for upper respiratory tract infection. Two behavioural antecedents were differentially targeted: self-efficacy from SCT and anticipated consequences from OLT (also represented in SCT as "risk perception").

Our theoretical framework included the constructs from three psychological models of behaviour that have been well validated in non-health professional populations: TPB [14], SCT [15], OLT [16] and the post-intention conceptual model of Implementation Intentions [24]. Like Eccles et al [23], we found that each of these models were predictive of GP intention to manage URTI without prescribing antibiotics and their simulated behaviour, although such relationships are not inherent in OLT and for II, the proposal is that II follow from BI. The variance explained by each model and overall was very similar to that observed in the previous predictive study [23], adding further support for the utility of these models in this approach. We found that each intervention had a significant effect on its targeted theoretical construct and that one intervention also improved the behavioural intention and simulated behaviour of GPs in relation to managing URTI without antibiotics.

GPs receiving the Graded Task intervention reported greater confidence in their ability to manage uncomplicated URTI without prescribing antibiotics than those who did not receive this intervention. This intervention also showed a significant effect on one of the TPB perceived behavioural control constructs (PBC Indirect). In a similar way to the self-efficacy construct, PBC describes the extent to which the individual feels they have personal control over a given behaviour. It is encouraging that this intervention changed only those constructs relating to control and suggests that this intervention is having its effect as we would predict based on the underlying rationale for its development. This intervention did not, however, have a measurable impact on either behavioural intention or behavioural simulation. Self-efficacy was selected as a target construct as it was found by Eccles *et al.*[23] to be a significant predictor of behavioural intention, behavioural simulation and actual behaviour. As intention is not a feature of SCT there is no theoretical basis to expect self-efficacy to have a direct effect on intention. It is still theoretically possible, however, that the intervention targeting self-efficacy might affect actual behaviour as both the TPB and SCT propose that control cognitions (PBC and self-efficacy) have a direct effect on behaviour, not mediated by behavioural intention.

GPs receiving the Persuasive Communication intervention had stronger intentions to manage URTI without prescribing antibiotics and were less likely to prescribe antibiotics than those who did not. They also reported greater anticipation of positive consequences for themselves and their patients in managing URTI without prescribing antibiotics than those receiving no intervention and those receiving the Graded Task intervention. This is an interesting finding in that it suggests that previous communications relating to the prescribing of antibiotics for the management of URTI (eg clinical guidelines) have not persuaded some GPs of the potential positive consequences of managing URTI symptomatically.

We further found that the effect of this intervention on both outcome measures was mediated through the targeted construct (anticipated consequences) and through a number of none targeted constructs: TPB attitude and subjective norm (latter mediated the effect on intention only) and SCT self-efficacy. This analysis suggests that the constructs provided by the theoretical frameworks we employed were behaving as proposed by their respective theories, supporting the value of these theories to guide the design and evaluation of interventions to change professional practice. The Persuasive Communication also showed a significant effect on two additional theoretical constructs not directly targeted by this intervention. Scores on the measure of habit (OLT) and one of the implementation intention (II) items (prior planning) were significantly enhanced even though they were not directly targeted by this intervention. The effect of the intervention on either behavioural intention or behavioural simulation was not mediated through either of these constructs. This possibly reflects the fact that the GPs taking part in the evaluation already had a strong habit to manage patients with URTI symptomatically and also had well developed plans of what they would do to achieve this.

### Strength and limitations

A significant strength of this study is the experimental design. Experimental modelling and manipulation of key theoretical constructs provided insight into the potential influence of these variables on clinical behaviour, using a method that is both robust and replicable. The response rates achieved are both a strength and a limitation of this study. A limitation is the quite low response rate for the initial survey (just over 32%). However, the recruited and randomised sample represented GP responses from 66% of the primary care practices we sampled, was of equal proportions across 13 PCTs and did not differ from the wider sampling frame on any of the demographic variables measured. In addition, the post-intervention retention rate was high (around 86%). The validity of the study findings is further strengthened by a high level of participant engagement with the study interventions

One aim of this research approach is to develop a replicable methodology for the design, evaluation and refinement of interventions before conducting a costly service level assessment – an intervention modelling process. In this study, the theoretical interim endpoints of behavioural intention and behavioural simulation were used as proxy measures for clinicians' actual behaviour. For this method to be reliable, such proxy measures of behaviour must be predictive of actual behaviour. While there is evidence to suggest that behavioural intention is a reliable proxy for actual clinical behaviour, the validity of other frequently used proxy measures of behaviour – like patient vignettes – is less well established. The measure of simulated behaviour was self-reported but the variance explained in the present study (10% and 14%) is closer to that reported in studies examining the relationship between behavioural intention and observed behaviour. In designing the format of the scenario presentation, we tried to replicate the real world setting as near as possible. The scenario presentation style was designed to replicate element s of routine consultations – for example we adopted the layout of the computer screen commonly seen by GPs in the surgery and used pictures of an actual prescription pad. Previous studies have presented patient scenarios as a block of descriptive text. It is possible that the introduction of visual elements increased saliency. We also asked GPs to note their diagnosis and management of each fictional patient rather than give a simple "Yes/No" response to indicate their decision to prescribe or not. Again this may have further increased their engagement with the task and encouraged them to respond in a way more closely representative of their actual behaviour.

## Conclusion

Understanding why and how interventions have their effect will allow implementation researchers to choose and develop more targeted behaviour change strategies, and have greater confidence in their effectiveness when applied at the service-level. The systematic approach used in this and our partner paper for the development and evaluation of interventions to change professional behaviour describes an intervention modelling process that corresponds closely to the theoretical and modelling phases recommended by the MRC Framework. We have shown the feasibility and value of this process in the development and evaluation of two behaviour change interventions. The theoretical framework provides an understanding of how and why these interventions differentially impacted on behaviour, offering the basis for the development of a taxonomy of intervention components. The approach presented in these partner papers promotes the development of professional behaviour change interventions that are replicable, are underpinned by a robust explanatory scientific rationale and that use generalisable elements of behaviour.

## Competing interests

The author(s) declare that they have no competing interests.

## Authors' contributions

All authors contributed to the conception and design of the study and approved the submitted draft.

## Pre-publication history

The pre-publication history for this paper can be accessed here:



## Supplementary Material

Additional file 1 Study questionnaire booklet. Questionnaire booklet containing theory-based items as presented to participants Click here for file

Additional file 2 The graded task intervention. A copy of the paper-based graded task intervention as presented to participants. Click here for file

Additional file 3 The persuasive communication intervention. A copy of the paper-based persuasive communication intervention as presented to participants. Click here for file
