# Supplementary material for: An intervention modelling experiment to change GPs' intentions to implement evidence-based practice: using theory-based interventions to promote GP management of upper respiratory tract infection without prescribing antibiotics #2
Source: BMC Health Serv Res. 2008 Jan 14;8:10. doi: 10.1186/1472-6963-8-10 (PMC2262061; doi:10.1186/1472-6963-8-10)
Supplement: Additional file 1 — Study questionnaire booklet. Questionnaire booklet containing theory-based items as presented to participants [file 1472-6963-8-10-S1.doc]

*Confidential*

#
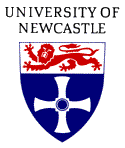


# Better understanding clinical practice:

# The management of URTIs

Thank you very much for participating in Part 2 of this project. As before, this questionnaire refers to factors influencing the management of patients, presenting for the first time, with an upper respiratory tract infection (URTI) in general practice.

For the purpose of this questionnaire, URTI includes sore throat, nasal discharge and coughs.

This questionnaire has TWO sections. The first contains a series of clinical management questions and the second a number of case scenarios.

It will take you between 15 and 30 minutes to complete the full booklet.

Please complete both sections.

#
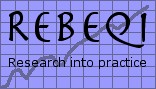


**1.02**

**/**

# SECTION ONE

**Most questions are answered by circling one number; a few require more time to answer.**

**Some questions are worded very similarly but they are different and it is important for the study that you answer them all.**

**Please try not to take too long over each response as we would like to know your immediate views and experiences.**

**Your answers are completely confidential.**

**1** **From memory, approximately how many of the last 10 patients who presented with an URTI for the first**

**time, did you manage without** prescribing an antibiotic

| 0 | 1 | 2 | 3 | 4 | 5 | 6 | 7 | 8 | 9 | 10 |
| --- | --- | --- | --- | --- | --- | --- | --- | --- | --- | --- |

**2** **I feel under pressure to manage patients with URTIs**

**without prescribing an antibiotic: *Strongly Strongly***

***disagree agree***

| a) from patients | 1 | 2 | 3 | 4 | 5 | 6 | 7 |
| --- | --- | --- | --- | --- | --- | --- | --- |
| b) from secondary care colleagues | 1 | 2 | 3 | 4 | 5 | 6 | 7 |
| c) from colleagues within the practice | 1 | 2 | 3 | 4 | 5 | 6 | 7 |
| d) from feedback provided by PACT | 1 | 2 | 3 | 4 | 5 | 6 | 7 |
| e) from published literature | 1 | 2 | 3 | 4 | 5 | 6 | 7 |

**3** **In general, managing a patient with an URTI without**

**prescribing an antibiotic would: *Strongly Strongly***

***disagree agree***

| a) Reassure them | 1 | 2 | 3 | 4 | 5 | 6 | 7 |
| --- | --- | --- | --- | --- | --- | --- | --- |
| b) Alleviate their symptoms | 1 | 2 | 3 | 4 | 5 | 6 | 7 |
| c) Increase their satisfaction with my care | 1 | 2 | 3 | 4 | 5 | 6 | 7 |
| d) Make them less likely to expect an antibiotic for a URTI in the future | 1 | 2 | 3 | 4 | 5 | 6 | 7 |
| e) Mean that the patient will re-consult for the same URTI episode | 1 | 2 | 3 | 4 | 5 | 6 | 7 |
| f) Increase the time taken for their URTI to resolve | 1 | 2 | 3 | 4 | 5 | 6 | 7 |
| g) Reduce the length of the consultation | 1 | 2 | 3 | 4 | 5 | 6 | 7 |
| h) Decrease the likelihood of antibiotic resistance in the community  i) Mean that the patient will consult with a different doctor  in subsequent episodes | 1  1 | 2  2 | 3  3 | 4  4 | 5  5 | 6  6 | 7  7 |

**4.** **If I routinely manage patients with URTIs without**

**prescribing antibiotics then: *Strongly Strongly***

***disagree agree***

| a) On balance, my life as a GP will be easier in the long run | 1 | 2 | 3 | 4 | 5 | 6 | 7 |
| --- | --- | --- | --- | --- | --- | --- | --- |
| b) On balance, the consequences for me as a GP (eg stress, time, future consultations etc) will be worse in the long run | 1 | 2 | 3 | 4 | 5 | 6 | 7 |

***Strongly Strongly***

***disagree agree***

| **5 It is highly likely that patients with an URTI will be worse off if I manage them without prescribing an antibiotic** | 1 | 2 | 3 | 4 | 5 | 6 | 7 |
| --- | --- | --- | --- | --- | --- | --- | --- |

**6** **How confident are you in your ability:**

# Not at all Extremely confident confident

| a) To manage patients with URTIs without prescribing an  antibiotic? | 1 | 2 | 3 | 4 | 5 | 6 | 7 |
| --- | --- | --- | --- | --- | --- | --- | --- |
| b) To end a consultation for a patient with an URTI who you have managed without prescribing an antibiotic? | 1 | 2 | 3 | 4 | 5 | 6 | 7 |
| c) To manage a patient whose URTI symptoms are distressing to them, without prescribing an antibiotic? | 1 | 2 | 3 | 4 | 5 | 6 | 7 |

***Strongly Strongly***

***disagree agree***

| **7** a) When I see patients with URTIs, I automatically consider managing them without prescribing an antibiotic | 1 | 2 | 3 | 4 | 5 | 6 | 7 |
| --- | --- | --- | --- | --- | --- | --- | --- |
| b) It is my usual practice to manage patients with URTIs without prescribing antibiotics | 1 | 2 | 3 | 4 | 5 | 6 | 7 |
| c) I aim to manage patients with URTIs without prescribing  an antibiotic | 1 | 2 | 3 | 4 | 5 | 6 | 7 |

1. **Given 10 patients presenting for the first time with an URTI, how many patients would you intend to manage without prescribing an antibiotic?**

| 0 | 1 | 2 | 3 | 4 | 5 | 6 | 7 | 8 | 9 | 10 |
| --- | --- | --- | --- | --- | --- | --- | --- | --- | --- | --- |

**9** **I find it difficult to manage patients presenting with an**

**URTI without prescribing an antibiotic who: *Strongly Strongly***

***disagree agree***

| a) Have already tried to self medicate for an URTI | 1 | 2 | 3 | 4 | 5 | 6 | 7 |
| --- | --- | --- | --- | --- | --- | --- | --- |
| b) Expect me to prescribe an antibiotic | 1 | 2 | 3 | 4 | 5 | 6 | 7 |
| c) Have a past history of an Chronic Obstructive Airways Disease | 1 | 2 | 3 | 4 | 5 | 6 | 7 |

**10** **Generally I find it difficult:  *Strongly Strongly***

***disagree agree***

| a) To manage patients with URTIs without prescribing an antibiotic | 1 | 2 | 3 | 4 | 5 | 6 | 7 |
| --- | --- | --- | --- | --- | --- | --- | --- |
| b) To end a consultation for a patient with an URTI who I have managed without prescribing an antibiotic | 1 | 2 | 3 | 4 | 5 | 6 | 7 |
| c) To manage a patient whose URTI symptoms are distressing to them without prescribing an antibiotic | 1 | 2 | 3 | 4 | 5 | 6 | 7 |

***Strongly Strongly***

***disagree agree***

| **11** a) I would like to manage patients with URTIs without prescribing an antibiotic, but I don’t really know if I can | 1 | 2 | 3 | 4 | 5 | 6 | 7 |
| --- | --- | --- | --- | --- | --- | --- | --- |
| b) Whether I manage patients with an URTI without prescribing antibiotics is entirely up to me | 1 | 2 | 3 | 4 | 5 | 6 | 7 |
| c) I am confident that I can manage patients with URTIs without prescribing an antibiotic whenever I want to | 1 | 2 | 3 | 4 | 5 | 6 | 7 |
| d) I can overcome all obstacles, whatever they may be, in managing an URTI without prescribing an antibiotic | 1 | 2 | 3 | 4 | 5 | 6 | 7 |

**12** **In general**: ***Strongly Strongly***

***disagree agree***

| a) The benefits of managing patients with URTIs without prescribing antibiotics outweigh the harms | 1 | 2 | 3 | 4 | 5 | 6 | 7 |
| --- | --- | --- | --- | --- | --- | --- | --- |
| b) Managing patients with URTIs without prescribing antibiotics is more often bad practice than good | 1 | 2 | 3 | 4 | 5 | 6 | 7 |
| c) Managing patients with URTIs without prescribing antibiotics is more often unsatisfying than satisfying | 1 | 2 | 3 | 4 | 5 | 6 | 7 |

**13** **In general:**

# Unimportant Important

| a) Reassuring patients is | 1 | 2 | 3 | 4 | 5 | 6 | 7 |
| --- | --- | --- | --- | --- | --- | --- | --- |
| b) Alleviating patient symptoms is | 1 | 2 | 3 | 4 | 5 | 6 | 7 |
| c) Increasing patient satisfaction with my care is | 1 | 2 | 3 | 4 | 5 | 6 | 7 |
| d) Reducing their expectation of an antibiotic for a URTI in the future is | 1 | 2 | 3 | 4 | 5 | 6 | 7 |
| e) Reducing the likelihood that the patient will consult again for the same URTI episode is | 1 | 2 | 3 | 4 | 5 | 6 | 7 |
| f) Reducing the time taken for a patient’s URTI to resolve is | 1 | 2 | 3 | 4 | 5 | 6 | 7 |
| g) Reducing the length of the consultations for URTIs is | 1 | 2 | 3 | 4 | 5 | 6 | 7 |
| h) Reducing antibiotic resistance is | 1 | 2 | 3 | 4 | 5 | 6 | 7 |

# 14 How motivated are you to do what: Not at all Very much

| a) Patients think you should | 1 | 2 | 3 | 4 | 5 | 6 | 7 |
| --- | --- | --- | --- | --- | --- | --- | --- |
| b) Secondary care colleagues think you should | 1 | 2 | 3 | 4 | 5 | 6 | 7 |
| c) Colleagues in primary care think you should | 1 | 2 | 3 | 4 | 5 | 6 | 7 |
| d) PACT feedback states that you should | 1 | 2 | 3 | 4 | 5 | 6 | 7 |
| e) The published literature states that you should | 1 | 2 | 3 | 4 | 5 | 6 | 7 |

# 15 Without an antibiotic, how confident are you in your ability to manage patients with URTIs who: Not at all Extremely confident confident

| a) Have already tried to self medicate for an URTI | 1 | 2 | 3 | 4 | 5 | 6 | 7 |
| --- | --- | --- | --- | --- | --- | --- | --- |
| b) Expect you to prescribe an antibiotic | 1 | 2 | 3 | 4 | 5 | 6 | 7 |
| c) Have a past history of Chronic Obstructive Airways Disease | 1 | 2 | 3 | 4 | 5 | 6 | 7 |

***Strongly Strongly***

***disagree agree***

| **16** **When a patient presents with an URTI, I have in mind to manage them without prescribing an antibiotic** | 1 | 2 | 3 | 4 | 5 | 6 | 7 |
| --- | --- | --- | --- | --- | --- | --- | --- |

| **17** **I intend to manage patients who present with an URTI without prescribing an antibiotic** | 1 | 2 | 3 | 4 | 5 | 6 | 7 |
| --- | --- | --- | --- | --- | --- | --- | --- |
| **18** **Currently my standard method of managing patients with an URTI involves managing them without prescribing an antibiotic** | 1 | 2 | 3 | 4 | 5 | 6 | 7 |

***Strongly Strongly***

disagree agree

| **19 I have a clear plan of:**  **a) how I will manage patients with an URTI without**  **prescribing an antibiotic.**  **b) when I will manage patients with an URTI without**  **prescribing an antibiotic.**  **c) under what circumstances I will manage patients with**  **an URTI without prescribing an antibiotic.** | 1  1  1 | 2  2  2 | 3  3  3 | 4  4  4 | 5  5  5 | 6  6  6 | 7  7  7 |
| --- | --- | --- | --- | --- | --- | --- | --- |

**20 If you have a plan, could you please describe it:** ____________________________________________________________________________________________________________________________________________________________________________________________________________________________________________________________________________________________________________________________________________________

SECTION TWO

The following pages contain a series of scenarios which include elements that may influence your management of patients presenting for the first time with an URTI.

**We would like you to consider each scenario in the context of a routine morning surgery.**

**You have 8 patients to see and two routine house calls pending.**

**It is February and there hasn’t been an influenza epidemic**

We appreciate the observational and communication skills you may normally draw on during an actual consultation cannot be a factor in your decision.

**Please try to consider each scenario based on the information presented, then, in the space provided, record your decisions relating to:**

- Diagnosis
- Management
- How difficult it was for you to decide your management of each scenario.

**A worked example is provided on the opposite page.**

**WORKED EXAMPLE**

**No. 000. Master Adam Simpson, 23 George Street, Othertown Age 8yrs**

**Clinical Records**

**A** Add  **X** All non-values **C** Consultations **P** Problems **J** IOS Claims

**V** Values  **I** Immunisations **M** Medications  **N** Investigations **L** Patient notes

**H** Health **T** Templates  **F** Forms & Admin **B** Allergies **Q** More

Active Problems : Nil Smoker : Not recorded

Significant past : Nil Occupation : Not recorded

Current medication : Nil

*HISTORY* : **3 days cough, sore throat++, fever.**

*EXAMINATION:* **Red pharynx, tonsils enlarged, tonsilar nodes ++, ears NAD**

**1.** Write your **diagnosis** here

**2.** Write your **management decision** here.

**3.** If you decide to **prescribe drugs** please write this on the script as below

**
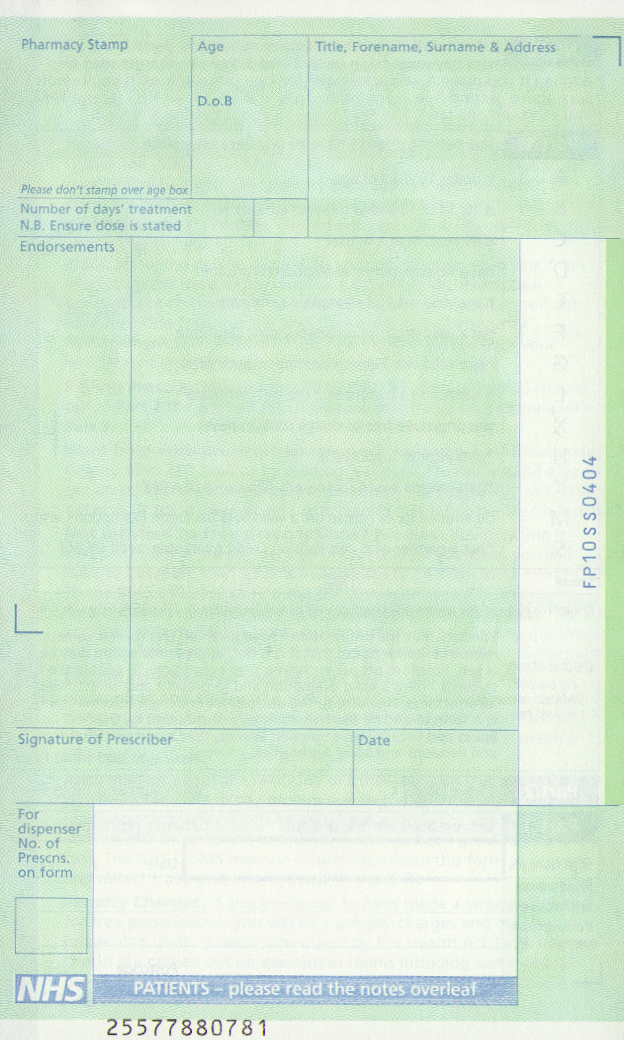
**

**DIAGNOSIS:**______**”*Tonsillitis*”**__________________

______________________________________________

______________________________________________

______________________________________________

***“Brand X Antibiotic 3x daily”***

***“Paracetamol 250mg/5ml, 5ml qds prn, 100ml”***

______________________________________________

______________________________________________

**MANAGEMENT**: ________________________________

_______***”Advised bed rest & plenty fluids”***____

______________________________________________

______________________________________________

**4.** Finally, please indicate below **how difficult** it was for you to make your decision about this scenario

______________________________________________

____________________________________________

______________________________________________

______________________________________________

**On the scale 1 to 10, how difficult was it for you to make a decision for this scenario?**

| **Not at all difficult** | 0 | 1 | 2 | 3 | 4 | 5 | 6 | 7 | 8 | 9 | 10 | **Extremely difficult** |
| --- | --- | --- | --- | --- | --- | --- | --- | --- | --- | --- | --- | --- |

*If you wish to comment on this decision please do so here*

*____________________________________________________________________________________________*

_____________________________________________________________________________________

_____________________________________________________________________________________

**No. 001 Miss Melody Dent, 2 Burnside Mews, Othertown Age 7 years**

**Clinical Records**

**A** Add  **X** All non-values **C** Consultations **P** Problems **J** IOS Claims

**V** Values  **I** Immunisations **M** Medications  **N** Investigations **L** Patient notes

**H** Health **T** Templates  **F** Forms & Admin **B** Allergies **Q** More

**Active Problems:** Nil  **Smoker:** Not recorded

**Significant past :** Otitis media 1999 **Occupation:** Not recorded

Otitis media 2000 x3

Otitis media 2003 x2

**Current medication:** Nil

*HISTORY*: ***4 days, sore throat, runny nose and earache***

*EXAMINATION:* ***Red pharynx, nodes ++, tympanic membranes injected***

**
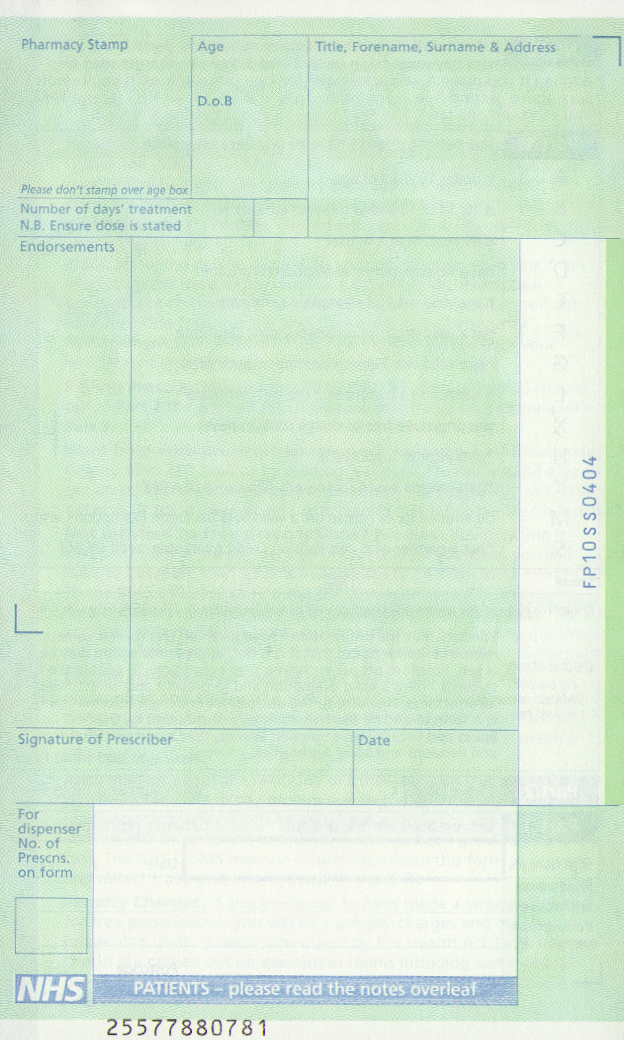
**

**DIAGNOSIS:___________________________________**

______________________________________________

______________________________________________

______________________________________________

______________________________________________

______________________________________________

**MANAGEMENT**: ________________________________

______________________________________________

______________________________________________

______________________________________________

______________________________________________

____________________________________________

______________________________________________

______________________________________________

**On the scale 1 to 10, how difficult was it for you to make a decision for this scenario?**

| **Not at all difficult** | 0 | 1 | 2 | 3 | 4 | 5 | 6 | 7 | 8 | 9 | 10 | **Extremely difficult** |
| --- | --- | --- | --- | --- | --- | --- | --- | --- | --- | --- | --- | --- |

*If you wish to comment on this decision please do so here*

*____________________________________________________________________________________________*

_____________________________________________________________________________________

_____________________________________________________________________________________

**No. 002. Mr James Armstrong, 10 Chapel Close, Othertown Age 62 years**

**Clinical Records**

**A** Add  **X** All non-values **C** Consultations **P** Problems **J** IOS Claims

**V** Values  **I** Immunisations **M** Medications  **N** Investigations **L** Patient notes

**H** Health **T** Templates  **F** Forms & Admin **B** Allergies **Q** More

**Active Problems:** Nil  **Smoker:** 15-20 / day

**Significant past :** Prostatectomy 2003  **Occupation:** Unemployed (ex-miner)

Osteoarthritis (knees) 2000

COPD 1998

Appendicitis 1963

**Current medication:** Temazepam

*HISTORY*: ***Sore throat 10 days, cough, yellowish phlegm***

*EXAMINATION:* ***Red pharynx, no nodes, chest clear***

**
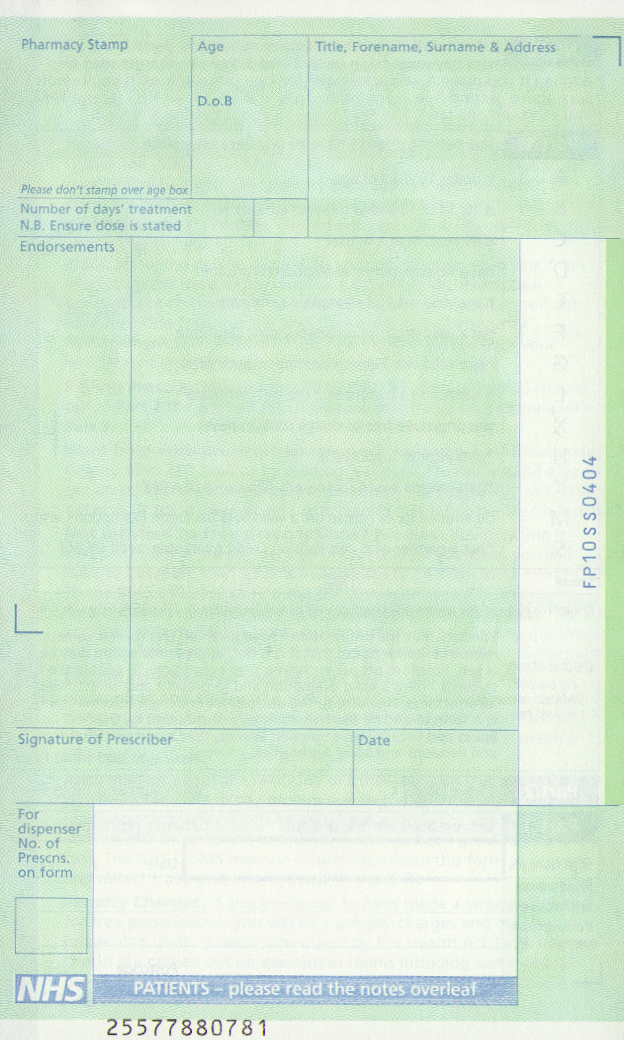
**

**DIAGNOSIS:___________________________________**

______________________________________________

______________________________________________

______________________________________________

______________________________________________

______________________________________________

**MANAGEMENT**: ________________________________

______________________________________________

______________________________________________

______________________________________________

______________________________________________

____________________________________________

______________________________________________

______________________________________________

**On the scale 1 to 10, how difficult was it for you to make a decision for this scenario?**

| **Not at all difficult** | 0 | 1 | 2 | 3 | 4 | 5 | 6 | 7 | 8 | 9 | 10 | **Extremely difficult** |
| --- | --- | --- | --- | --- | --- | --- | --- | --- | --- | --- | --- | --- |

*If you wish to comment on this decision please do so here*

*____________________________________________________________________________________________*

_____________________________________________________________________________________

_____________________________________________________________________________________

**No. 003. Miss Sarah Mathers, 5 Sycamore Avenue, Othertown Age 4 months**

**Clinical Records**

**A** Add  **X** All non-values **C** Consultations **P** Problems **J** IOS Claims

**V** Values  **I** Immunisations **M** Medications  **N** Investigations **L** Patient notes

**H** Health **T** Templates  **F** Forms & Admin **B** Allergies **Q** More

**Active Problems:** Nil  **Smoker:** Not recorded

**Significant past :** Nil **Occupation:** Not recorded

**Current medication:** Nil

*HISTORY*: ***2 days, cough, fever, unwell, off feeds.***

***Older sister with similar just now – “she got antibiotics”***

*EXAMINATION:* ***Crying, ears normal (limited view), throat red, chest clear***

**
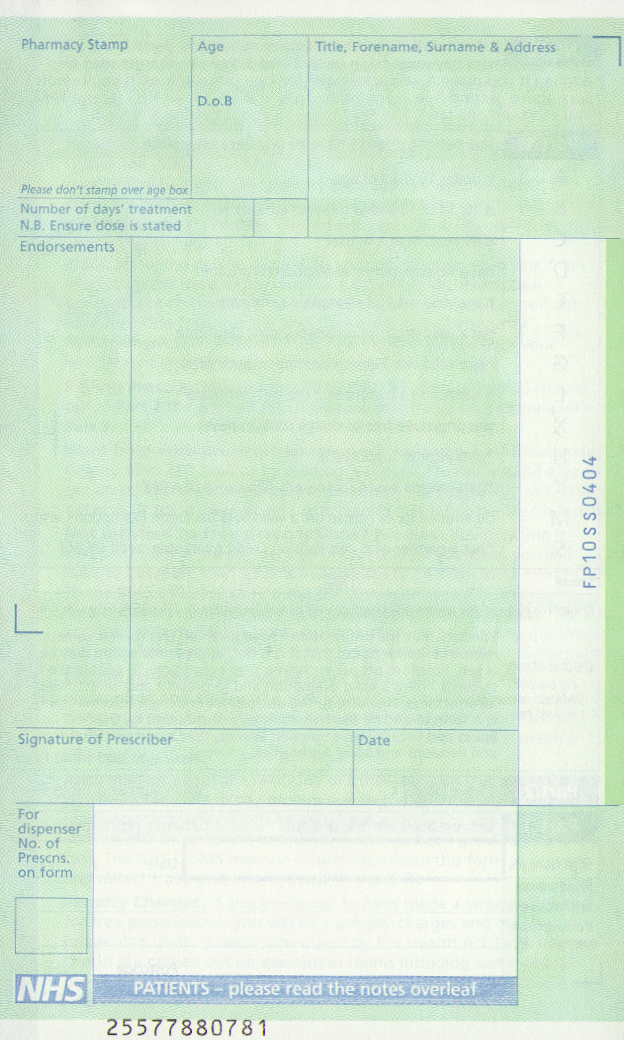
**

**DIAGNOSIS:___________________________________**

______________________________________________

______________________________________________

______________________________________________

______________________________________________

______________________________________________

**MANAGEMENT**: ________________________________

______________________________________________

______________________________________________

______________________________________________

______________________________________________

____________________________________________

______________________________________________

______________________________________________

**On the scale 1 to 10, how difficult was it for you to make a decision for this scenario?**

| **Not at all difficult** | 0 | 1 | 2 | 3 | 4 | 5 | 6 | 7 | 8 | 9 | 10 | **Extremely difficult** |
| --- | --- | --- | --- | --- | --- | --- | --- | --- | --- | --- | --- | --- |

*If you wish to comment on this decision please do so here*

*____________________________________________________________________________________________*

_____________________________________________________________________________________

_____________________________________________________________________________________

**No. 004. Miss Aman Naseer, 16 Mitchell Street, Othertown Age 23 years**

**Clinical Records**

**A** Add  **X** All non-values **C** Consultations **P** Problems **J** IOS Claims

**V** Values  **I** Immunisations **M** Medications  **N** Investigations **L** Patient notes

**H** Health **T** Templates  **F** Forms & Admin **B** Allergies **Q** More

**Active Problems:** Nil  **Smoker:** Not recorded

**Significant past :** Nil  **Occupation:** Not recorded

**Current medication:** Nil

*HISTORY*: ***Sore throat, 9 days now. Wants antibiotic to clear it***

*EXAMINATION:* ***Red pharynx, nodes ++***

**
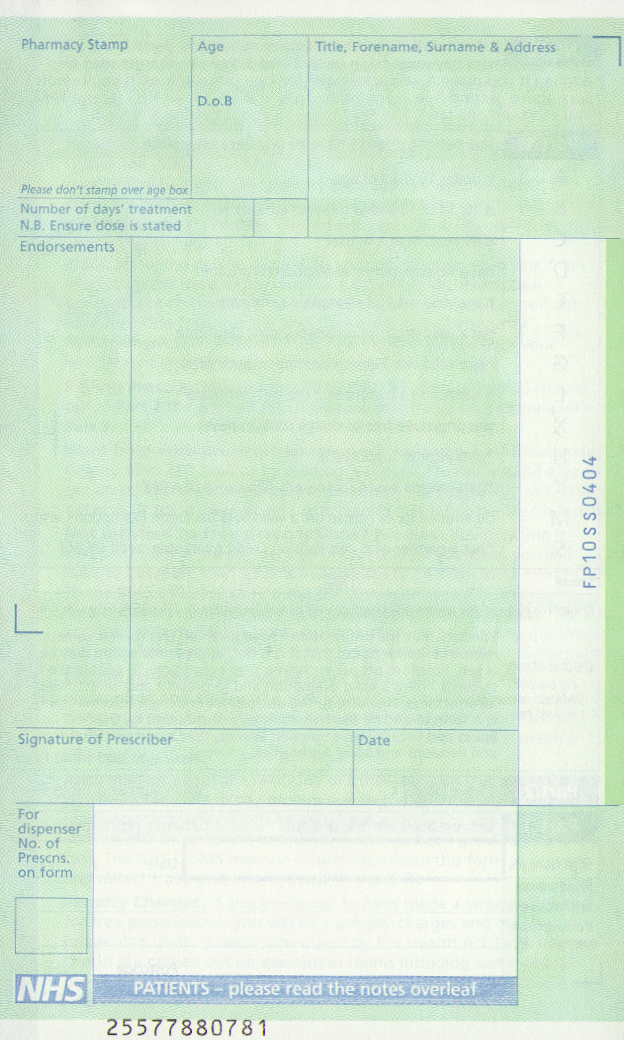
**

**DIAGNOSIS:___________________________________**

______________________________________________

______________________________________________

______________________________________________

______________________________________________

______________________________________________

**MANAGEMENT**: ________________________________

______________________________________________

______________________________________________

______________________________________________

______________________________________________

____________________________________________

______________________________________________

______________________________________________

**On the scale 1 to 10, how difficult was it for you to make a decision for this scenario?**

| **Not at all difficult** | 0 | 1 | 2 | 3 | 4 | 5 | 6 | 7 | 8 | 9 | 10 | **Extremely difficult** |
| --- | --- | --- | --- | --- | --- | --- | --- | --- | --- | --- | --- | --- |

*If you wish to comment on this decision please do so here*

*____________________________________________________________________________________________*

_____________________________________________________________________________________

_____________________________________________________________________________________

**No. 005. Mrs Lisa Saunders, 17 Northcote Avenue, Othertown Age 34 years**

**Clinical Records**

**A** Add  **X** All non-values **C** Consultations **P** Problems **J** IOS Claims

**V** Values  **I** Immunisations **M** Medications  **N** Investigations **L** Patient notes

**H** Health **T** Templates  **F** Forms & Admin **B** Allergies **Q** More

**Active Problems:** Nil  **Smoker:** non-smoker

**Significant past :** Depression 1995  **Occupation:** Checkout operator

RTA 1993

Asthma 1981

**Current medication:** Salbutamol inhaler

*HISTORY*: ***5 days cough, phlegm (clear),sore throat ++, runny nose***

*EXAMINATION:* ***Chest clear, pus on tonsils, ears NAD, nodes ++***

**
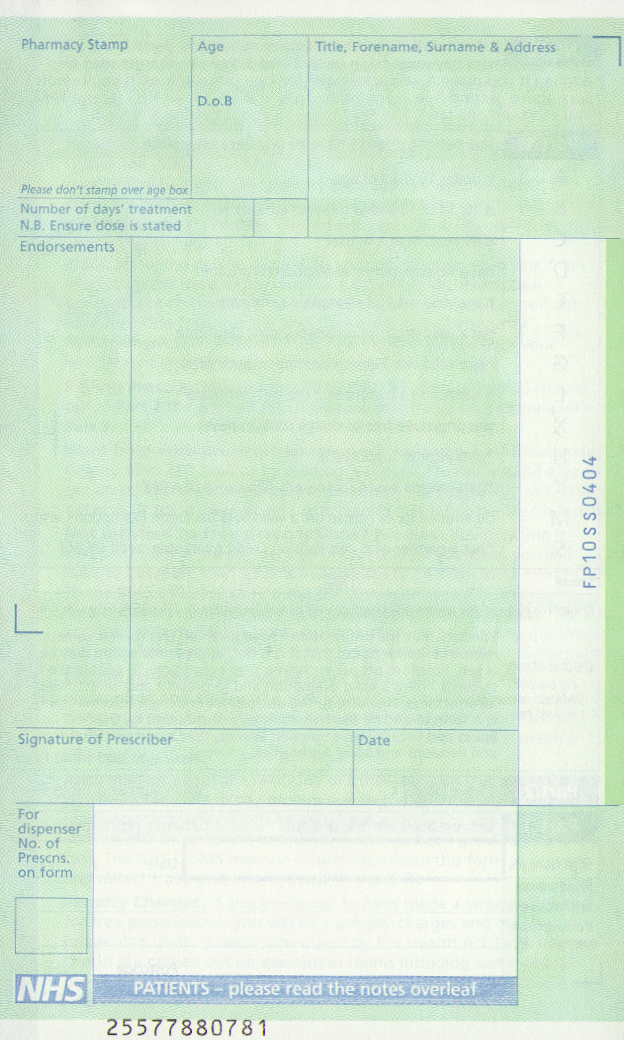
**

**DIAGNOSIS:___________________________________**

______________________________________________

______________________________________________

______________________________________________

______________________________________________

______________________________________________

**MANAGEMENT**: ________________________________

______________________________________________

______________________________________________

______________________________________________

______________________________________________

____________________________________________

______________________________________________

______________________________________________

**On the scale 1 to 10, how difficult was it for you to make a decision for this scenario?**

| **Not at all difficult** | 0 | 1 | 2 | 3 | 4 | 5 | 6 | 7 | 8 | 9 | 10 | **Extremely difficult** |
| --- | --- | --- | --- | --- | --- | --- | --- | --- | --- | --- | --- | --- |

*If you wish to comment on this decision please do so here*

*____________________________________________________________________________________________*

_____________________________________________________________________________________

_____________________________________________________________________________________

**No. 006. Master Owen Gallagher, 20 Ash Lane, Othertown Age 9 months**

**Clinical Records**

**A** Add  **X** All non-values **C** Consultations **P** Problems **J** IOS Claims

**V** Values  **I** Immunisations **M** Medications  **N** Investigations **L** Patient notes

**H** Health **T** Templates  **F** Forms & Admin **B** Allergies **Q** More

**Active Problems:** Nil  **Smoker:** Not recorded

**Significant past :** Nil  **Occupation:** Not recorded

**Current medication:** Nil

*HISTORY*: ***Unwell 3 days, fractious, crying all the time***

*EXAMINATION:* ***Responsive and alert, throat red, ears sl injected, Chest clear***

**
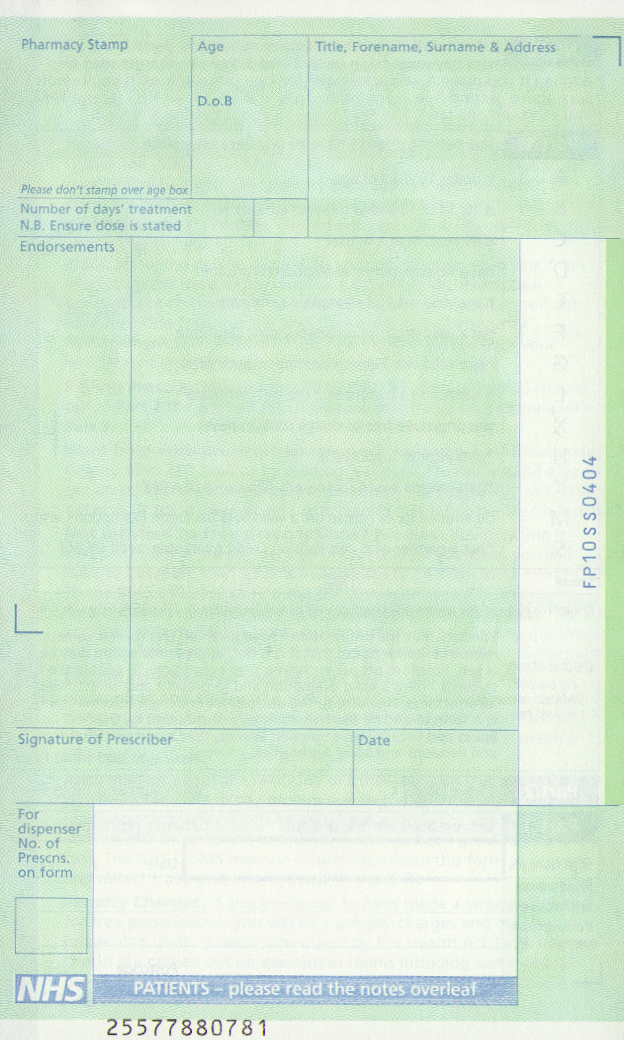
**

**DIAGNOSIS:___________________________________**

______________________________________________

______________________________________________

______________________________________________

______________________________________________

______________________________________________

**MANAGEMENT**: ________________________________

______________________________________________

______________________________________________

______________________________________________

______________________________________________

____________________________________________

______________________________________________

______________________________________________

**On the scale 1 to 10, how difficult was it for you to make a decision for this scenario?**

| **Not at all difficult** | 0 | 1 | 2 | 3 | 4 | 5 | 6 | 7 | 8 | 9 | 10 | **Extremely difficult** |
| --- | --- | --- | --- | --- | --- | --- | --- | --- | --- | --- | --- | --- |

*If you wish to comment on this decision please do so here*

*____________________________________________________________________________________________*

_____________________________________________________________________________________

_____________________________________________________________________________________

**No. 007. Mr Jarrod Burns, 58 St Thomas’ Drive, Othertown Age 19 years**

**Clinical Records**

**A** Add  **X** All non-values **C** Consultations **P** Problems **J** IOS Claims

**V** Values  **I** Immunisations **M** Medications  **N** Investigations **L** Patient notes

**H** Health **T** Templates  **F** Forms & Admin **B** Allergies **Q** More

**Active Problems:** Nil  **Smoker:** 20 / day

**Significant past :** RTA 2003  **Occupation:** Not recorded

**Current medication:** Nil

*HISTORY*: ***3 days sore throat+++, difficulty swallowing, “burning up”***

*EXAMINATION:* ***Tonsils enlarged, pus on tonsils, nodes +++***

**
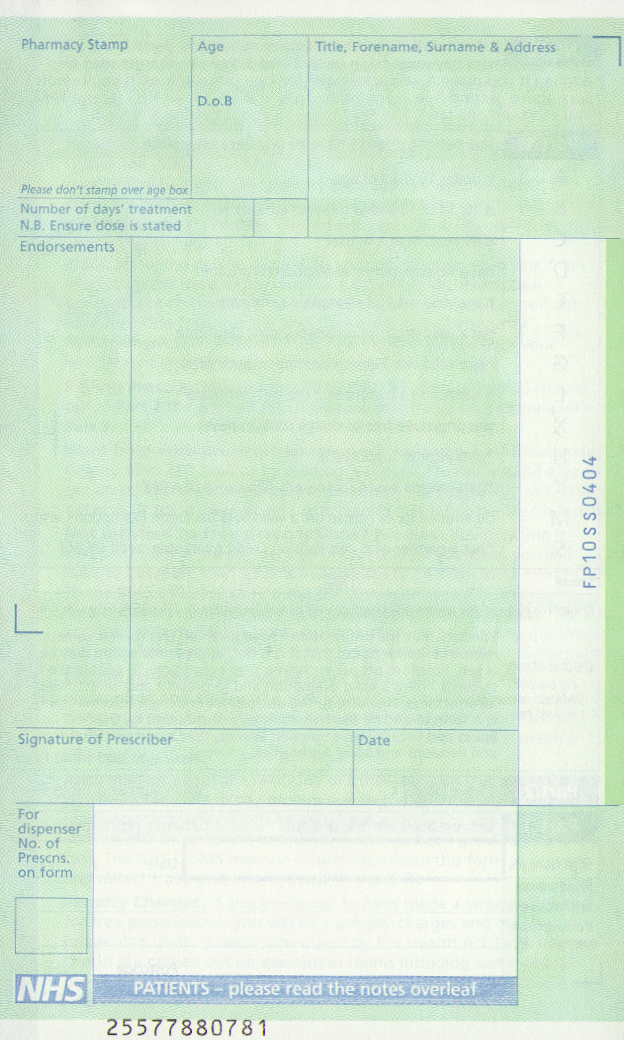
**

**DIAGNOSIS:___________________________________**

______________________________________________

______________________________________________

______________________________________________

______________________________________________

______________________________________________

**MANAGEMENT**: ________________________________

______________________________________________

______________________________________________

______________________________________________

______________________________________________

____________________________________________

______________________________________________

______________________________________________

**On the scale 1 to 10, how difficult was it for you to make a decision for this scenario?**

| **Not at all difficult** | 0 | 1 | 2 | 3 | 4 | 5 | 6 | 7 | 8 | 9 | 10 | **Extremely difficult** |
| --- | --- | --- | --- | --- | --- | --- | --- | --- | --- | --- | --- | --- |

*If you wish to comment on this decision please do so here*

*____________________________________________________________________________________________*

_____________________________________________________________________________________

_____________________________________________________________________________________

**No. 008 Miss Nada Abdul, 43 Moorside Crescent, Othertown Age 5 years**

**Clinical Records**

**A** Add  **X** All non-values **C** Consultations **P** Problems **J** IOS Claims

**V** Values  **I** Immunisations **M** Medications  **N** Investigations **L** Patient notes

**H** Health **T** Templates  **F** Forms & Admin **B** Allergies **Q** More

**Active Problems:** Nil  **Smoker:** Not recorded

**Significant past :** Eczema 2001 **Occupation:** Not recorded

**Current medication:** Aqueous cream

*HISTORY*: ***Sore throat 4 days, earache 2 days, cough***

*EXAMINATION:* ***Pus on tonsils, nodes ++, ears NAD, chest clear***

**
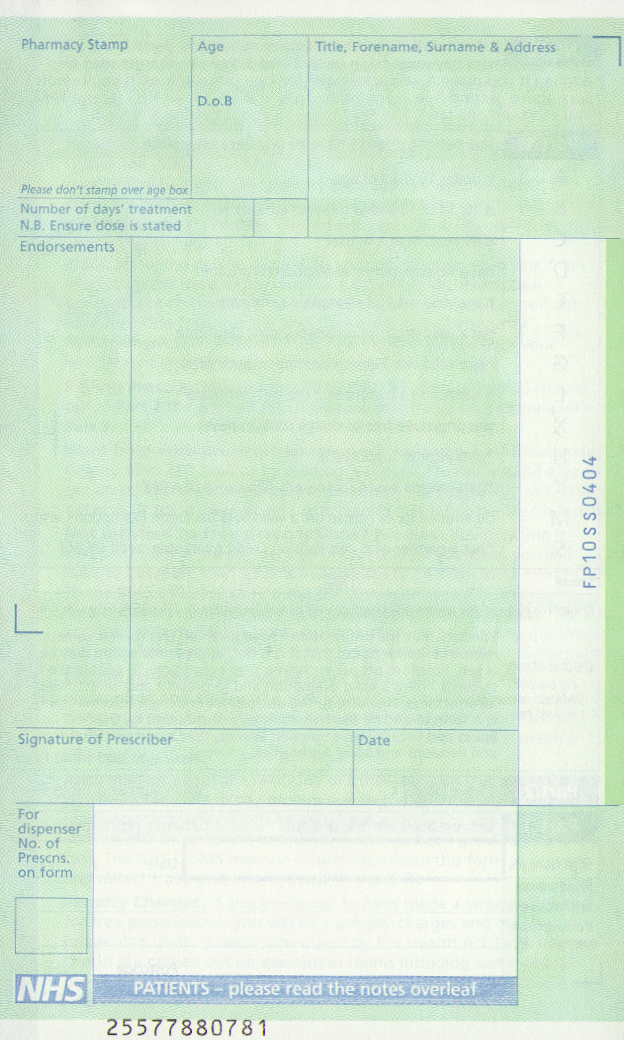
**

**DIAGNOSIS:___________________________________**

______________________________________________

______________________________________________

______________________________________________

______________________________________________

______________________________________________

**MANAGEMENT**: ________________________________

______________________________________________

______________________________________________

______________________________________________

______________________________________________

____________________________________________

______________________________________________

______________________________________________

**On the scale 1 to 10, how difficult was it for you to make a decision for this scenario?**

| **Not at all difficult** | 0 | 1 | 2 | 3 | 4 | 5 | 6 | 7 | 8 | 9 | 10 | **Extremely difficult** |
| --- | --- | --- | --- | --- | --- | --- | --- | --- | --- | --- | --- | --- |

*If you wish to comment on this decision please do so here*

*____________________________________________________________________________________________*

_____________________________________________________________________________________

_____________________________________________________________________________________

**Is there any other comment you would like to make?**

**________________________________________________________________________________________________________________________________________________________________________________________________________________________________________________________________________________________________________________________________**

**Thank you once again for your help with this study.**

**Please now return your completed questionnaire in the reply paid envelope provided**

If you wish to find out more about this study please contact:

Susan Hrisos

Research Associate

Centre for Health Services Research

University of Newcastle upon Tyne

21 Claremont Place

Newcastle upon Tyne

NE2 4AA

Tel: 0191 222 6774

Email: susan.hrisos@ncl.ac.uk
