# Supplementary material for: An intervention modelling experiment to change GPs' intentions to implement evidence-based practice: using theory-based interventions to promote GP management of upper respiratory tract infection without prescribing antibiotics #2
Source: BMC Health Serv Res. 2008 Jan 14;8:10. doi: 10.1186/1472-6963-8-10 (PMC2262061; doi:10.1186/1472-6963-8-10)
Supplement: Additional file 2 — The graded task intervention. A copy of the paper-based graded task intervention as presented to participants. [file 1472-6963-8-10-S2.doc]

# [Graded Task Intervention]

**SECTION ONE**

**Task 1**

**A).** **The following is a list of five situations relating to the management of sore throat.** The situations have been ranked from easiest to most difficult based upon the experience of fellow GPs of the management of URTIs.

**Starting with number 1, consider each situation in turn.** Place a **tick in the box on the right** to indicate how confident you are that you could achieve each situation.

***Could you confidently:***

| **YES** | **NO** | **MAYBE** |
| --- | --- | --- |
|  |  |  |
|  |  |  |
|  |  |  |
|  |  |  |
|  |  |  |

1. End a consultation for a patient with an URTI without prescribing an antibiotic

2. Manage patients with URTIs, without an antibiotic, who have already tried to self medicate for an URTI

3. Manage patients with URTIs, without an antibiotic, who expect you to prescribe an antibiotic

4. Manage patients with URTIs, without an antibiotic, whose symptoms are distressing them

5. Manage patients with URTIs, without an antibiotic, who have a past history of chronic obstructive

airways disease

**B).**

i) **If** **ALL** **your responses are YES,** can you think of a situation in which you **would** find it difficult to manage a patient with URTI without an antibiotic? Please describe this situation below and **then GO TO Part C:** _____________________________________________________________________________________________________________________________________________________________________________________________________________________________________________________________________

ii) **If you have responded NO or MAYBE** to any or all of the five situations above, select FROM THEM the situation which you find the LEAST difficult.

Write the number of your chosen situation here (1 – 5) and **then GO TO Part C**

**Part C is about your chosen situation.**

**C).** Imagine yourself with a patient in the situation you have described or selected above.

Make a list of all possible alternative management strategies for that situation.

_________________________________________________________________________________________________________________________________________________________________________________________________________________________________________________________________________________________________________________________________________________________________________________________________

Thinking about the strategy or strategies that you think would be most clinically appropriate and feasible, make a plan of what you will actually do to manage your chosen situation in the future. Write your plan below:

_________________________________________________________________________________________________________________________________________________________________________________________________________________________________________________________________________________________________________________________________________________________________________________________________
