# Supplementary material for: An intervention modelling experiment to change GPs' intentions to implement evidence-based practice: using theory-based interventions to promote GP management of upper respiratory tract infection without prescribing antibiotics #2
Source: BMC Health Serv Res. 2008 Jan 14;8:10. doi: 10.1186/1472-6963-8-10 (PMC2262061; doi:10.1186/1472-6963-8-10)
Supplement: Additional file 3 — The persuasive communication intervention. A copy of the paper-based persuasive communication intervention as presented to participants. [file 1472-6963-8-10-S3.doc]

**[Persuasive Communication intervention]**

Primary care practice involves a large range of clinical decisions, often made under pressure of time. These decisions have consequences not only for the immediate clinical condition of the patient but also for the way patients perceive the effectiveness of treatments and the role of the GP in their ongoing care.

Below is a series of pictorial scenarios, depicting some of the consequences for the GP of deciding to manage URTIs with or without antibiotics.

**Dr A,** in the first row of scenarios, **manages URTIs by prescribing antibiotics**, while **Dr B,** in the second row of scenarios, **manages URTIs**

**without prescribing antibiotics.**

Beneath the scenarios are questions to help you to consider the possible **consequences of each doctor’s prescribing habits**.

**Dr A** **manages patients with URTI by prescribing antibiotics**


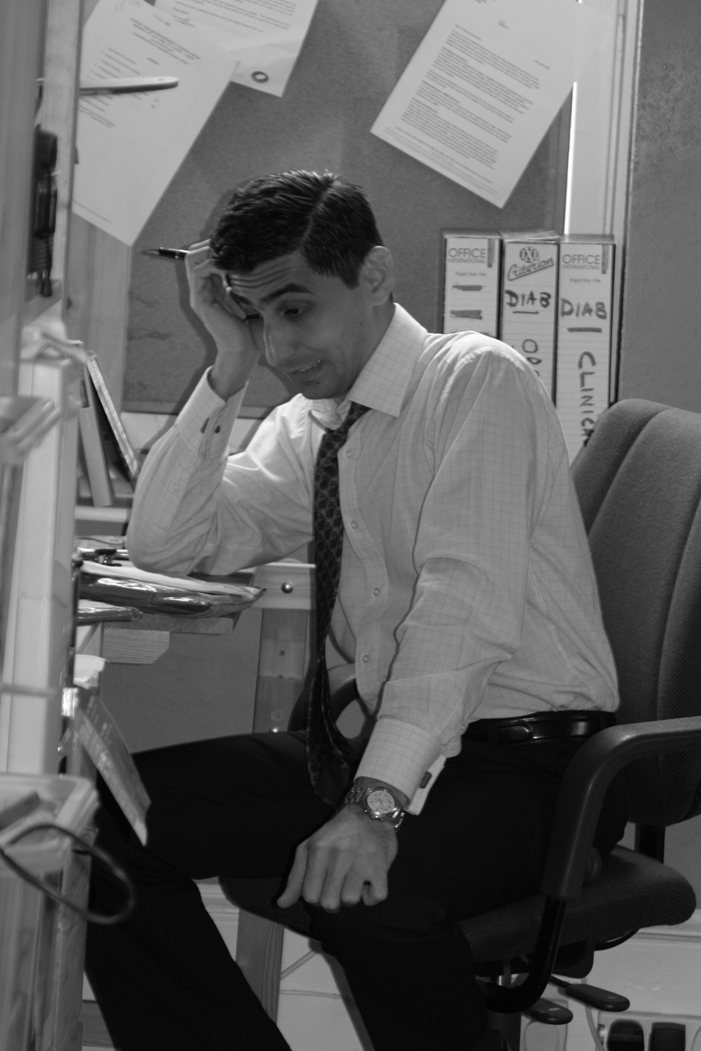

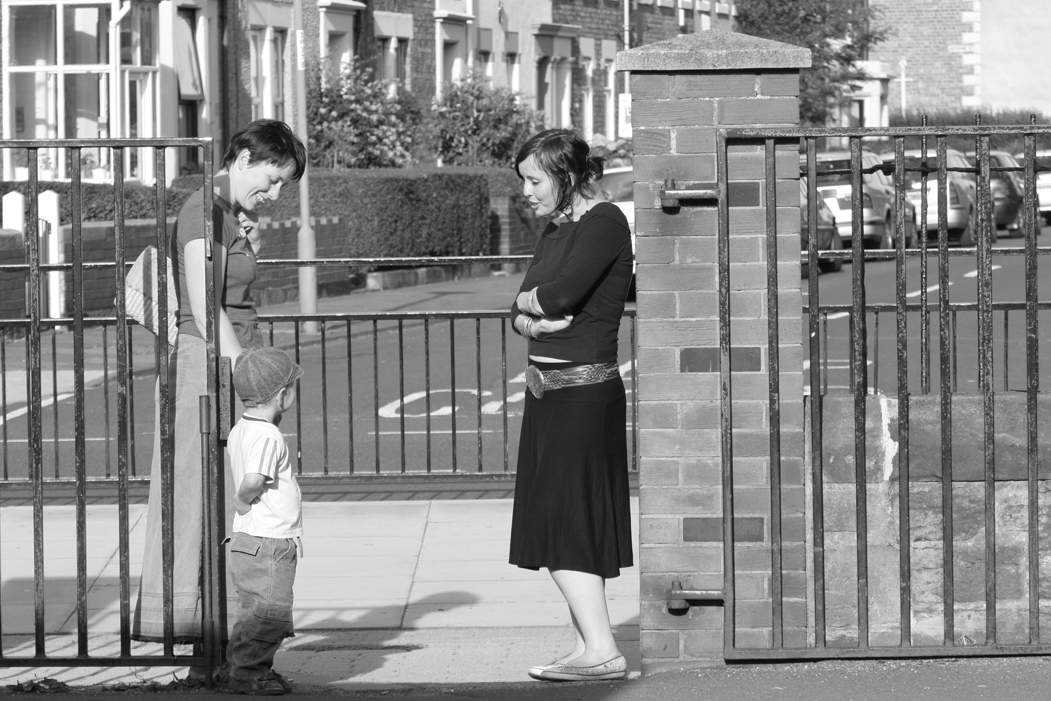


“I’m worried about our Colin, he’s got a dreadful cough and a sore throat.”

“You should take him to Dr A for some antibiotics.”


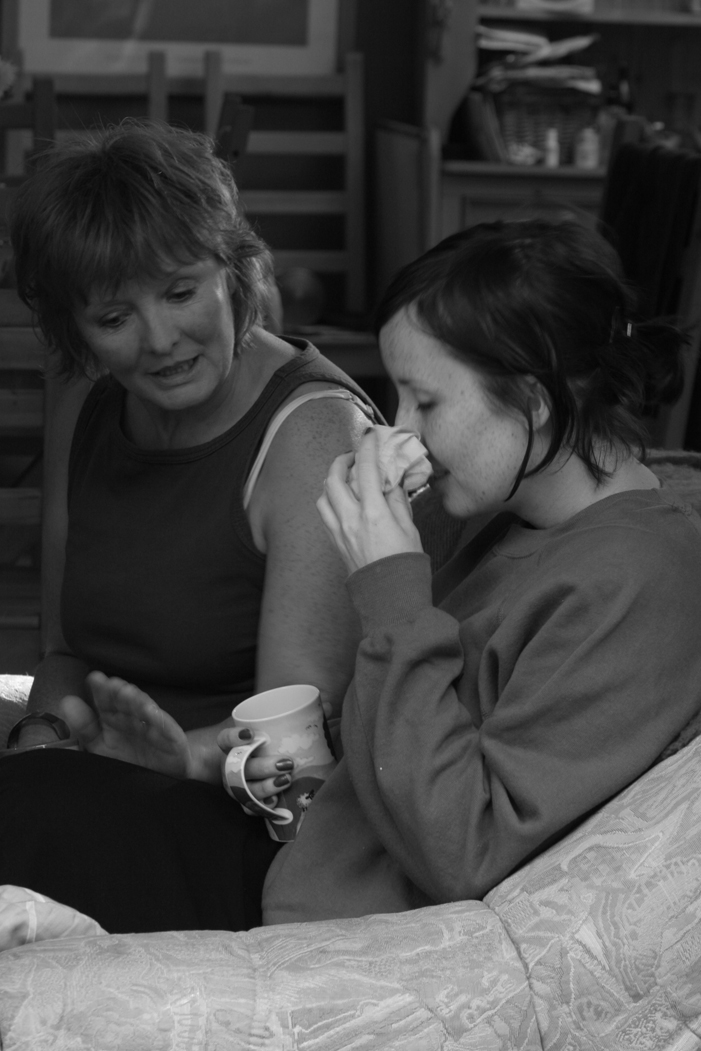


“I’m sorry I gave you my cold, here, have some of the antibiotics Dr A gave me.”


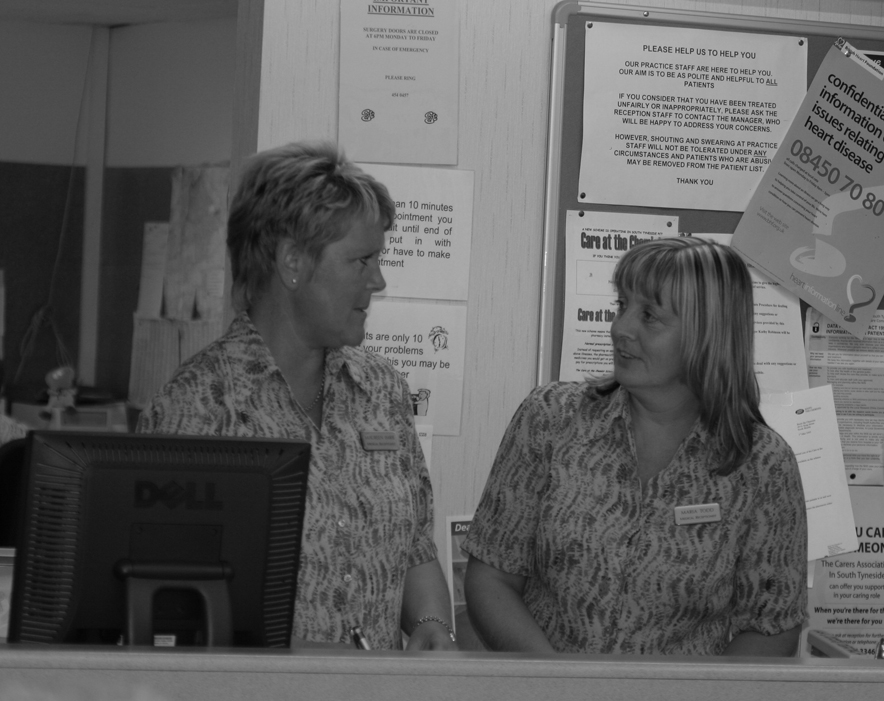


“More sore throats – does Dr A have any appointments left for this week?”

“Not another four extra’s with sore throats wanting antibiotics!”


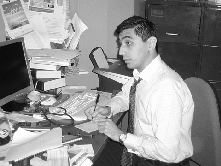


Penicillin 3 times daily

**Dr B** **manages patients with URTI symptomatically**


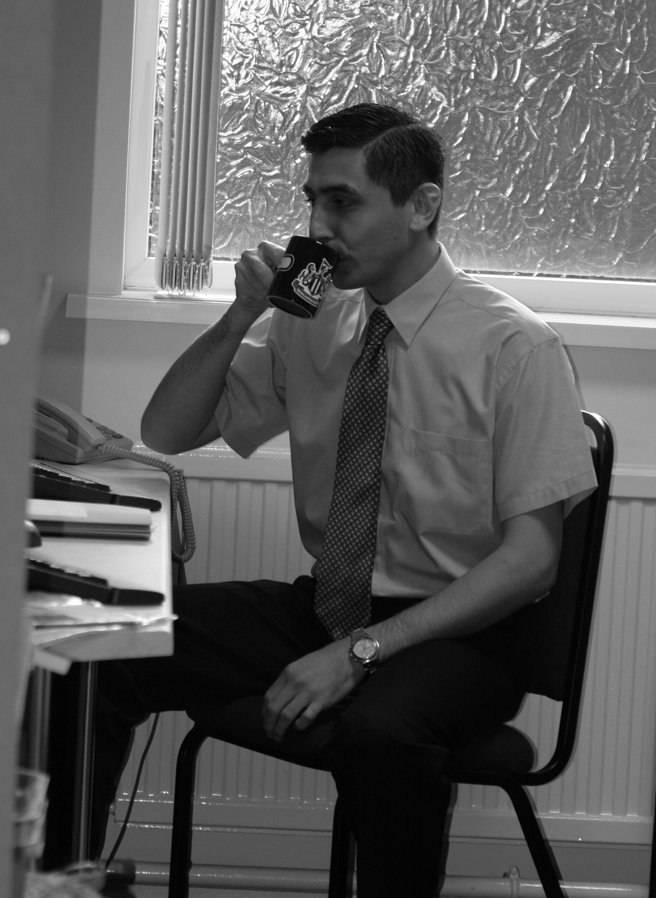

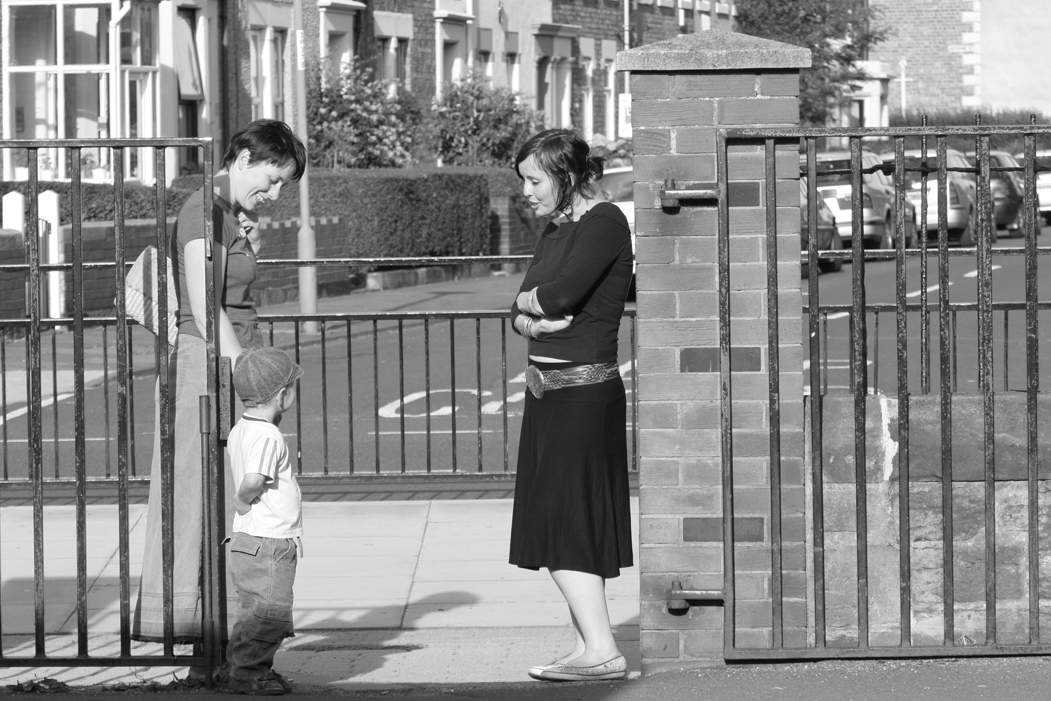


“I’m worried about our Colin, he’s got a dreadful cough and a sore throat.”

“Our Martin had that last week. A couple of days of Calpol and he was fine.”


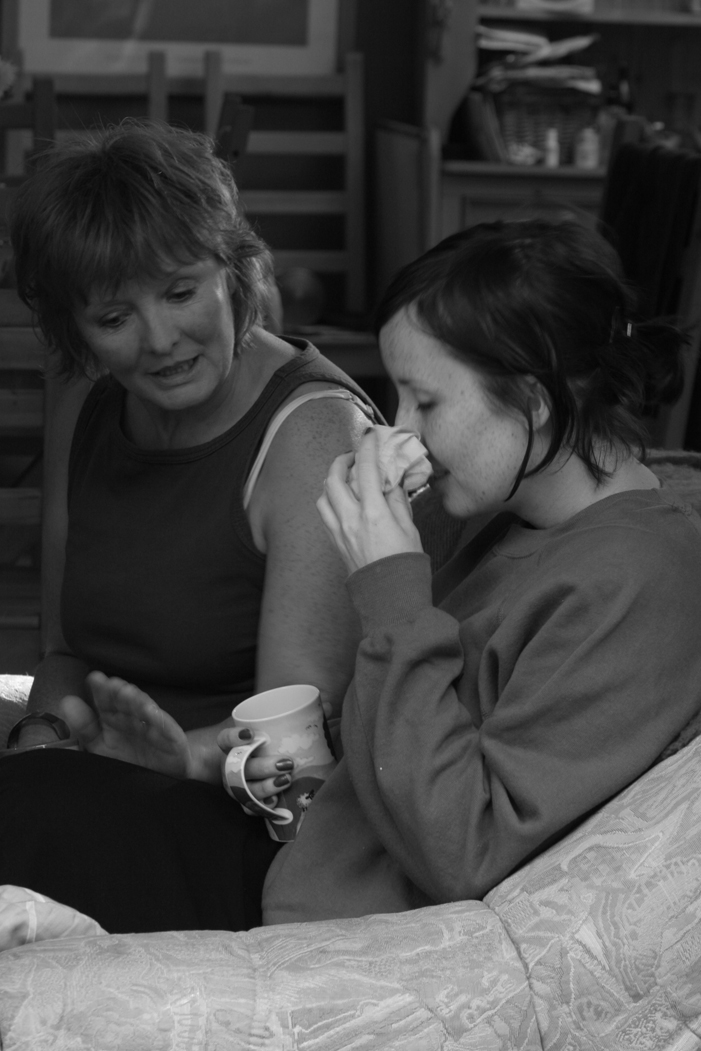


“I’m sorry I gave you my cold, here, let me get you some paracetamol.”


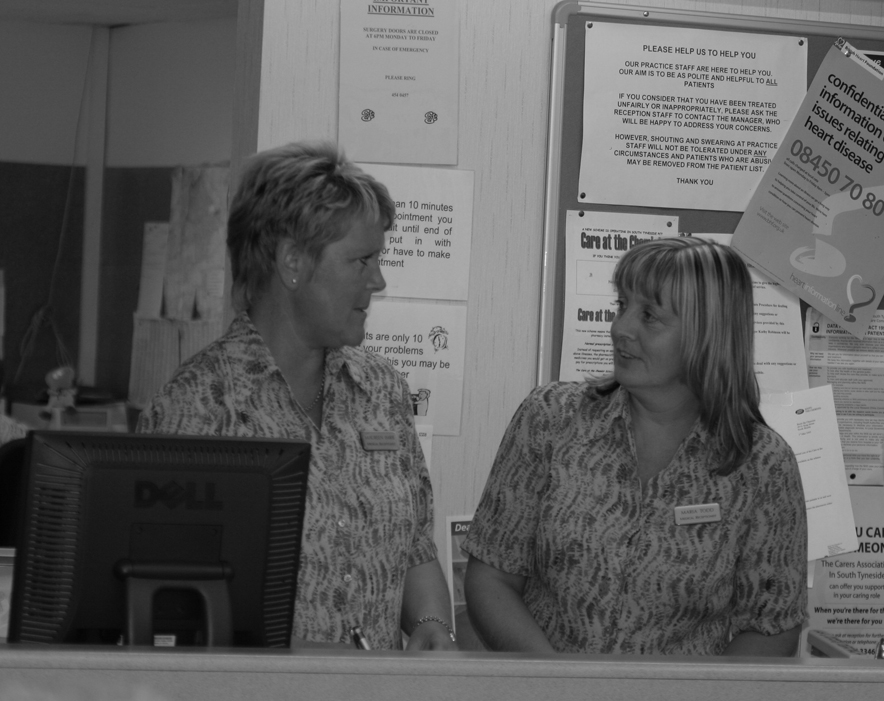


“That’s another ‘flu vac clinic booked up.”

“No extra’s today. I’ll enjoy this cup of tea before I start my visits!”


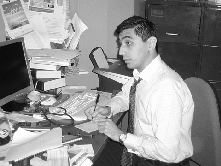


Paracetamol, fluids, bed rest

Whose patients have more Whose practice is more likely to Whose patients are more On balance, who has

expectation of getting antibiotics? have opportunities for prevention? likely to share antibiotics? the easier life?

**With respect to** **managing patients with URTI:**

Who do you try to be like?

***100% like Dr A* *100% like Dr B***

Who are you actually like?

***100% like Dr A* *100% like Dr B***

**Please now complete Section Two of the questionnaire**
